# Enhancement of *Nicotiana tabacum* Resistance Against Dehydration-Induced Leaf Senescence via Metabolite/Phytohormone-Gene Regulatory Networks Modulated by Melatonin

**DOI:** 10.3389/fpls.2021.686062

**Published:** 2021-07-06

**Authors:** Zheng Chen, Wei Jia, Songwei Li, Jiayang Xu, Zicheng Xu

**Affiliations:** ^1^College of Tobacco Science, Henan Agricultural University, Zhengzhou, China; ^2^College of Resources and Environmental Sciences, Henan Agricultural University, Zhengzhou, China

**Keywords:** dehydration stress, melatonin, reactive oxygen species, multi-omics, hormone profiling, carotenoid

## Abstract

Melatonin (MEL) is a pleiotropic agent with crucial functions reported in a variety of stress responses and developmental processes. Although MEL involvement in plant defense against natural leaf senescence has been widely reported, the precise regulatory mechanisms by which it delays stress-induced senescence remain unclear. In this study, we found that foliar spraying of melatonin markedly ameliorated dehydration-induced leaf senescence in *Nicotiana tabacum*, accompanied by attenuated oxidative damage, expression of senescence-related genes, and reduced endogenous ABA production. Metabolite profiling indicated that melatonin-treated plants accumulated higher concentrations of sugars, sugar alcohol, and organic acids, but fewer concentrations of amino acids in the leaves, than untreated plants after exposure to dehydration. Gene expression analysis revealed that the delayed senescence of stressed plants achieved by melatonin treatment might be partially ascribed to the upregulated expression of genes involved in ROS scavenging, chlorophyll biosynthesis, photosynthesis, and carbon/nitrogen balances, and downregulated expression of senescence-associated genes. Furthermore, hormone responses showed an extensively modulated expression, complemented by carotenoid biosynthesis regulation to achieve growth acceleration in melatonin-treated plants upon exposure to dehydration stress. These findings may provide more comprehensive insights into the role of melatonin in alleviating leaf senescence and enhancing dehydration resistance.

## Introduction

Drought is one of the most prominent environmental limitations restricting agricultural sustainability and food security worldwide (Tardieu et al., 2018). As sessile organisms, plants are inevitably challenged to detect and respond to water deprivation to mitigate negative effects on their development and yield (Antoniou et al., 2017). Drought stress triggers excessive accumulation of reactive oxygen species (ROS), which can cause oxidative damage to DNA, proteins, membrane lipids, and chlorophyll, leading to an imbalance in cellular metabolism (Corpas and Barroso, 2013; Sun et al., 2018). This redox imbalance decreases the photosynthetic performance of plants by interfering with the chloroplast structure and photosystem reaction centers (Shao et al., 2016; Sharma et al., 2020). Thus, improving the ability of crop plants to cope with reduced water availability has emerged as one of the most urgent aims for maintaining agricultural productivity under climate change conditions.

Plants have evolved highly sophisticated strategies to mediate the detrimental effects of drought, such as stomatal closure to decrease transpiration rates and accumulation of osmoprotectants and antioxidants to cope with ROS imbalance (Mundada et al., 2021; Wang et al., 2021). Particularly, during water deficit, the abscisic acid (ABA) production of plants is markedly increased and activates a complex signaling network that induces massive reprogramming of ABA-dependent gene expression, subsequently modulating cellular and physiological acclimation responses (Zhu, 2016; Kuromori et al., 2018). Additionally, plant tolerance to drought involves complex regulatory networks that control global gene expression, protein modification, and metabolite composition (Urano et al., 2010; Kim et al., 2017). At the transcriptional level, several different types of transcription factors (TFs), such as bHLH, bZIP, MYB, dehydration-responsive element-binding (DREB), and WRKY families, regulate drought responses by activating downstream defense genes (Sakuraba et al., 2015; Wang et al., 2020; Xiang et al., 2020). These adaptation responses constitute a critical part of the core advantages in sustaining plant survival under water-deficit conditions, although other unidentified metabolic adjustment could also be involved in stressed plant survival. However, if stress conditions are prolonged, the internal defense system of plants is insufficient to regulate ROS balance, ultimately causing oxidative damage to cellular constituents (Lee et al., 2012).

As the final and inevitable stage of leaf development, leaf senescence is controlled by highly coordinated action at the cell, tissue, organ, and organism levels (Lin et al., 2015). Plants initiate leaf senescence to facilitate the remobilization of nutrients and energy from senescing leaves into growing organs and optimize growth fitness at the end of the growing season (Zhang et al., 2018; Jalil et al., 2019), which is a developmental process mediated by aging and can also be triggered by unfavorable environmental conditions, such as nitrogen limitation, darkness, excessive light, high salinity, and desiccation (Yang et al., 2015; Zhao et al., 2016; Kamranfar et al., 2018). Moreover, leaf senescence is promoted by multiple plant hormones, such as ABA, ethylene, salicylic acid (SA), and jasmonic acid (JA), but repressed by cytokinin (Gan and Amasino, 1995; Cutler et al., 2010; Jiang et al., 2014; An et al., 2019). Stress-induced leaf senescence affects the extended photosynthesis duration and ultimately compromises plant growth and development. For example, drought stress results in premature leaf senescence, characterized by a reduction in chlorophyll synthesis owing to chloroplast disassembly and membrane ion leakage (Ma et al., 2018). The senescence processes are often associated with the upregulated expression of genes for several senescence-associated enzymes, such as chlorophyllase, pheophytinase, and Chl-degrading peroxidase, and downregulated expression of photosynthetic genes, including RIBULOSE BISPHOSPHATE CARBOXYLASE SMALL CHAIN (RBCS) (Jalil et al., 2017; Ma et al., 2018; Zhang et al., 2018; He et al., 2021). In nature, accelerated leaf abscission and senescence are characteristics of drought tolerance because these processes contribute to the maintenance of water balance in the whole plant body, thereby improving the chance of plant survival under drought conditions (Pic et al., 2002). However, in crop species, leaf senescence has been linked to yield reduction because it decreases the accumulation of photosynthetic-assimilates. As transgenic plants with delayed leaf senescence have been shown to exhibit enhanced drought resistance associated with higher photosynthetic efficiency (Rivero et al., 2007), intervention of the senescence process by agents could confer dehydration stress tolerance in plants.

Melatonin (MEL, *N*-acetyl-5-methoxytryptamine), a well-known multifunctional biomolecule, was discovered over 60 years ago and has been widely applied in physiology and medicine (Reiter et al., 2014). In mammals, MEL exerts significant biological activities in regulating circadian rhythm, sleep–wake cycles, mood, sexual and reproductive behavior, seasonal photoperiod, immunological system, and detoxification of free radicals (Hernández-Ruiz et al., 2005; Calvo et al., 2013; Shi et al., 2015a). Plant MEL was initially identified in 1995 and has shown great potential in the regulation of plant growth, immunity, phytoremediation, and plant–rhizomicrobial interactions (Dubbels et al., 1995; Li et al., 2021). In the past 20 years, MEL has generated considerable interest, with hundreds of articles relating to MEL in plants published in PubMed (Supplementary Figure 1). As reviewed by Zhang et al. (2015), MEL biosynthesis in plants occurs through a biosynthesis pathway similar to that in animals, with four consecutive enzymes, including tryptophan decarboxylase (TDC), tryptamine 5-hydroxylase (T5H), arylalkylamine *N*-acetyltransferase (AANAT), and *N-acetylserotonin* methyltransferase (ASMT), identified to participate in MEL biosynthesis. In transgenic experiments, transgenic plants overexpressing ovine *AANAT* and ovine *ASMT* genes exhibited MEL levels significantly higher than that of the wild type (Park et al., 2013; Wang et al., 2014). In 2018, the first MEL plant receptor (CAND2/PMTR1) involved in MEL signaling perception was identified in *Arabidopsis* (Wei et al., 2018), and is thought to be a potential candidate phytohormone.

MEL is implicated in regulating various biological processes in plant systems, such as seed germination, growth, floral transition, rooting induction, photosynthesis, ripening/senescence, and cell protective responses (Arnao and Hernández-Ruiz, 2019). As an emerging plant growth regulator, MEL plays a prominent role in enhancing plant tolerance to stress stimuli, and at appropriate concentrations, most abiotic stresses causing oxidative damage could be alleviated by MEL (Arnao and Hernández-Ruiz, 2014). As several other classic antioxidants, MEL functions as a free radical scavenger to protect plants against a variety of biotic and abiotic stressors, such as pathogen infection, high salinity, alkalinity, extreme temperature, radiation, and heavy metal exposure (Yamamoto and Mohanan, 2001; Meng et al., 2014; Gong et al., 2017; Siddiqui et al., 2019, 2020; Li et al., 2021), and it can directly detoxify ROS and indirectly regulate oxidative homeostasis in plants by activating cellular enzymatic and non-enzymatic antioxidants (Arnao and Hernández-Ruiz, 2014). Furthermore, MEL has been shown to protect plants from acid rain (Debnath et al., 2018), paraquat (Wang et al., 2018), and senescence (Wang et al., 2013a), and herbicide-induced oxidative stress was significantly ameliorated in MEL-rich transgenic plants (Park et al., 2013). In recent years, MEL has been found to be effective in preserving chlorophyll content in several plants, including barley (Arnao and Hernández-Ruiz, 2009), perennial ryegrass (Zhang et al., 2016), and rice (Liang et al., 2015). Although tremendous progress has been achieved in unraveling MEL mitigation effects in plant responses to abiotic stress, little is known about the detailed mechanisms of leaf senescence regulation by MEL to enhance dehydration resistance.

Tobacco is not only a model plant, but also an economically influential crop worldwide. While tobacco’s development is highly susceptible to water stress, drought resulted in decreases of the biomass and reducing sugar content, and increases of total nitrogen and nicotine contents in flue-cured tobacco plants. Here, we studied the response of tobacco (*Nicotiana tabacum* L.) pretreated with MEL to long-term dehydration stress, with the aim of elucidating MEL-mediated metabolite/phytohormone-gene regulatory networks. To this end, an integrated analysis of the transcriptome and metabolome was employed to investigate the effects of external MEL application on dehydration-induced leaf senescence. The findings from this study not only provide a crucial therapeutic strategy to mitigate the extreme water deficit, but also reveal the protective role of MEL in repressing dehydration-induced leaf senescence.

## Materials and Methods

### Plant Materials and Treatments

**FIGURE 1:**
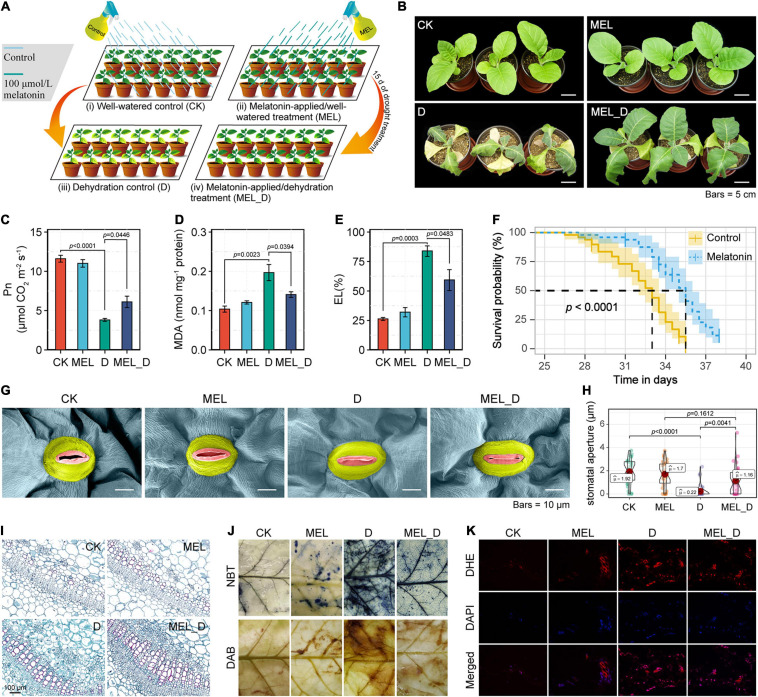
Mitigation effects of exogenous melatonin on dehydration stress in *Nicotiana tabacum* seedlings. **(A)** Diagrammatic representation of the experimental design. CK, well-watered control; MEL, melatonin (MEL)-applied/well-watered treatment; D, dehydration control; MEL_D, melatonin-applied/dehydration treatment. The schematic was created using Adobe Illustrator software. **(B)** Phenotypic effects of melatonin pretreatment on *N. tabacum* seedlings exposed to dehydration stress for 15 days. **(C–E)** Measurement of photosynthetic rate (Pn), MDA concentration, and electrolyte leakage (EL). Error bars represent the SE calculated from three independent biological replicates. **(F)** Survival analysis of control and melatonin (100 μmol)-pretreated 2-week-old seedlings subjected to prolonged drought stress, by withholding watering (*n* = 50). **(G)** Scanning electron microscope (SEM) images of stomata from the fourth leaves of plants at 15 days of dehydration treatment. **(H)** Statistical analysis of stomatal aperture. **(I)** Images of transverse sections of the stems of seedlings after 15 days of dehydration stress. Red indicates degree of lignification. **(J)** Histochemical staining of control and MEL treatment leaves exposed to dehydration stress for 15 days. **(K)** Fluorescence microscopic analysis of leaf ROS levels. Results of dihydroethidium (DHE) staining (upper), 4,6-diamidino-2-phenylindole (DAPI) staining (middle), and overlapping images (lower) have been presented.

Sterilized tobacco seeds were germinated in the dark and then transferred to pots containing a mixture of peat and vermiculite (1:1, v/v) for culture in a greenhouse, as previously described (Chen et al., 2019). For MEL treatment, half of the 3-week-old potted seedlings were sprayed with 100 μM MEL solution (10 ml) every day for 1 week. In a previous trial, this treatment was shown to be quite effective in alleviating abiotic stress (Ye et al., 2016; Wang et al., 2019). Prior to dehydration treatment, the seedlings were bottom-irrigated with Hoagland’s nutrient solution three times a week. After 4 weeks in the glasshouse, both control and MEL-treated plants were exposed to the cessation of watering. Dehydration stress was imposed by withholding watering for 15 days, when tobacco seedlings exhibited extensive phenotypic foliar injury (in the form of severe wilting and leaf chlorosis). In the experimental design (Figure 1A), healthy and uniform seedlings were subjected to four treatments: CK (non-MEL/well-watered treatment), MEL (100 μM MEL-applied/well-watered treatment), D (non-MEL/dehydration treatment), and MEL_D (100 μM MEL-applied/dehydration treatment). The fourth plant leaves were sampled at the indicated time points and kept at −80°C until further processing. Non-stressed controls were sampled at 4 weeks + 15 days in parallel with the drought treatment.

### Histochemical Assays

*In situ* accumulation of O_2_^–^ and H_2_O_2_ was visually detected using nitroblue tetrazolium (NBT) and diaminobenzidine (DAB) as substrates, respectively (Sen et al., 2017). Intracellular ROS levels were evaluated by dihydroethidium and then visualized using a Nikon Eclipse E600 epifluorescence microscope. For programmed cell death, the samples were assessed with a TUNEL apoptosis assay, as previously described (Li et al., 2018).

### Electron Microscopy

Observation of leaf stomata was performed using an SU8010 scanning electron microscope (SEM, Hitachi, Tokyo, Japan), and its aperture was determined using ImageJ software (National Institutes of Health, Bethesda, MD, United States). Additionally, leaf ultrastructure was observed and imaged at 80 kV using a Hitachi H-7650 transmission electron microscope.

### Physiological and Biochemical Detection

Relative water content (RWC) was measured following the method of Xiao et al. (2009), and photosynthetic rate (Pn) was determined as described by Huo et al. (2015). The maximal photochemical efficiency (*F*_v_/*F*_m_) was determined using a PAM-2100 Chl fluorometer (Walz, Effeltrich, Germany). Electrolyte leakage (EL) was determined according to Shi et al. (2012). Chlorophyll content was estimated as reported by Gang et al. (2010). Malondialdehyde (MDA) content, superoxide radicals (O_2_^–^), hydrogen peroxide (H_2_O_2_) levels, and the activities of antioxidant enzymes (SOD [E.C. 1.15.1.1], POD [E.C. 1.11.1.7], and CAT [E.C. 1.11.1.6]) were determined using the corresponding detection kits (Jiancheng Bioengineering Institute, Nanjing, China), according to the manufacturer’s instructions. Determination of ascorbic acid (AsA) and dehydroascorbate (DHA) levels was performed according to the method of Xing et al. (2019).

### Metabolome Analysis

Metabolite extracts were analyzed using a Vanquish UHPLC system (Thermo Fisher, United States) coupled with an Orbitrap Q Exactive series mass spectrometer (Thermo Fisher, United States). The raw data files generated by UHPLC-MS/MS were processed using the Compound Discoverer 3.1 (CD3.1, Thermo Fisher) to perform peak alignment, peak picking, and quantitation for each metabolite. The normalized data was used to predict the molecular formula based on additive ions, molecular ion peaks and fragment ions. And then peaks were matched with the mzCloud^1^ and ChemSpider^2^ database to obtained the accurate qualitative and relative quantitative results. We applied univariate analysis (*t*-test) to calculate the statistical significance (*P*-value). The metabolites with VIP > 1 and *P*-value < 0.05 and fold change ≥ 2 or FC ≤ 0.5 were considered to be differential metabolites. The functions of metabolites and metabolic pathways were studied using the KEGG database. The reproducibility of untargeted analysis was assessed using six biological replicates.

### Transcriptomic Analysis

A total amount of 1 μg RNA per sample was used as input material for the RNA sample preparations. Sequencing libraries were generated using NEBNext^®^ UltraTM RNA Library Prep Kit for Illumina^®^ (NEB, United States) following manufacturer’s protocol and index codes were added to attribute sequences to each sample. The clustering of the index-coded samples was performed on a cBot Cluster Generation System using TruSeq PE Cluster Kit v3-cBot-HS (Illumia) according to the manufacturer’s instructions. After cluster generation, the library preparations were sequenced using an Illumina Novaseq platform and 150 bp paired-end reads were generated. After removing the adapters and low-quality sequence reads from the raw reads by Trimmomatic, the clean reads were mapped to the tobacco genome^3^ using Hisat2 v2.0.5. The gene expression value was calculated as fragments per kilobase of transcript per million mapped reads (FPKM). Functional assignment of genes was conducted using Gene Ontology (GO) enrichment analysis. The reproducibility of the RNA-seq analysis was assessed using three biological replicates. The raw RNA-sequencing data were deposited in the NCBI database under the accession number PRJNA691642.

### qPCR Assay

Total RNA extraction and first-strand cDNA synthesis were performed as previously described (Xia et al., 2018). qPCR analysis was performed using a CFX96TM Real-Time PCR Detection System (Bio-Rad, Hercules, CA, United States) according to the manufacturer’s recommendation. The relative gene expression level was calculated using the 2^–ΔΔ*CT*^ method (Livak and Schmittgen, 2001), and the tobacco *actin* gene was used as an internal control. Primer sequences were listed in Supplementary Table 8.

### Detection of Plant Hormone Content

Fresh tobacco leaves (50 mg) were frozen in liquid nitrogen, ground into powder, and extracted with 1 mL methanol/water/formic acid (15:4:1, V/V/V). The combined extracts were evaporated to dryness under nitrogen gas stream, reconstituted in 100 μL 80% methanol (V/V), and filtered through 0.22 μm filter for further LC-MS analysis. The sample extracts were analyzed using an LC-ESI-MS/MS system (UHPLC, ExionLC^TM^ AD^4^; MS, Applied Biosystems 6500 Triple Quadrupole, see Text Footnote 4). AB 6500 + QTRAP^®^ LC-MS/MS System, equipped with an ESI Turbo Ion-Spray interface, operating in both positive and negative ion modes and controlled by Analyst 1.6 software (AB Sciex).

### Determination of Carotenoid Composition

Given that carotenoids are precursors of ABA synthesis, the carotenoid composition in the leaves of the four treatments was detected. Carotenoid extracts were analyzed using an LC-APCI-MS/MS system (UHPLC, ExionLC^TM^ AD; MS, Applied Biosystems 6500 Triple Quadrupole). A YMC C30 column (3 μm, 100 mm × 2 mm) was used for HPLC analysis. The experiments were performed at 28°C with a flow rate of 0.8 mL/min. The mobile phase consisted of solvent A, i.e., methanol: acetonitrile (1:3, v/v) containing 0.01% BHT and 0.1% formic acid, and solvent B (methyl tert-butyl ether with 0.01% BHT). The gradient program was as follows: 0% B (0–3 min), increased to 70% B (3–5 min), then increased to 95% B (5–9 min), and finally ramped back to 0% B (11–12 min). MS analysis was carried out using the API 6500 + Q TRAP LC-MS/MS System, equipped with an APCI Turbo Ion-Spray interface, operating in positive ion mode and controlled by Analyst 1.6.3 software (AB Sciex).

### Statistical Analysis

Statistical analyses were performed using the statistical software R (R version R-4.0.2). Physiological and biochemical measurements were subjected to one-way ANOVA, and then, significant differences between individual means were assessed using Tukey’s test. Three developmentally identical leaves were pooled for each biological replicate. At least nine tobacco plants are required for three biological replicates.

## Results

### Exogenous Application of Melatonin Improves Dehydration Stress Resistance in *Nicotiana tabacum*

Phenotypic analysis revealed that exogenous MEL improved the tolerance of tobacco plants to water deprivation (Figure 1B), which was supported by positive changes in Pn, RWC, and biomass accumulation (Figure 1C and Supplementary Figure 2). With water deprivation, MEL_D-treated plants experienced less cell membrane damage than D controls, as verified by MDA content and EL in MEL_D being lower than those in D (Figures 1D,E). MEL and CK treatments displayed no differences for all examined parameters. Moreover, drought survival analysis showed that 2-week-old seedlings pretreated with MEL displayed higher survival rates relative to untreated seedlings after long-term dehydration stress (Figure 1F). Scanning electron microscopy showed that the stoma area in D- or MEL_D-treated leaves was smaller than that in well-watered leaves, while the stomata of stressed leaves were almost closed (Figures 1G,H), indicating that both D and MEL_D treatments dramatically induced stomatal closure. Subsequent anatomical observations revealed that MEL accelerated lignin biosynthesis and Casparian strip formation in tobacco plants compared to those of non-treated stressed plants (Figure 1I and Supplementary Figure 2).

Additionally, O_2_^–^ and H_2_O_2_ were observed *in situ* using NBT and DAB staining, respectively, with CK- and MEL-treated leaves being similarly and lightly stained without water deprivation. The staining became darker with dehydration, but more intense staining was seen in D than in MEL_D (Figure 1J), which was further confirmed by quantitative measurement (Supplementary Figure 3). Consistently, fluorescence microscopic analysis of ROS level also showed ROS toxicity in MEL-pretreated leaves being lower than that in untreated leaves under dehydration conditions (Figure 1K). Furthermore, MEL treatment increased the expression of several stress-responsive genes (*NtSOD*, *NtCAT*, *NtAPX*, *NtGST*, *NtLTP1*, *NtRD29A*, *NtERD10C*, *NtERD10D*, *NtSAMDC*, and *NtSPSA*) (Supplementary Figure 3) and accumulated higher antioxidant levels (SOD, POD, CAT, AsA, and DHA) (Supplementary Figure 4) compared with those of non-pretreated stressed plants.

### Melatonin Ameliorates Dehydration-Induced Premature Senescence in *Nicotiana tabacum*

**FIGURE 2:**
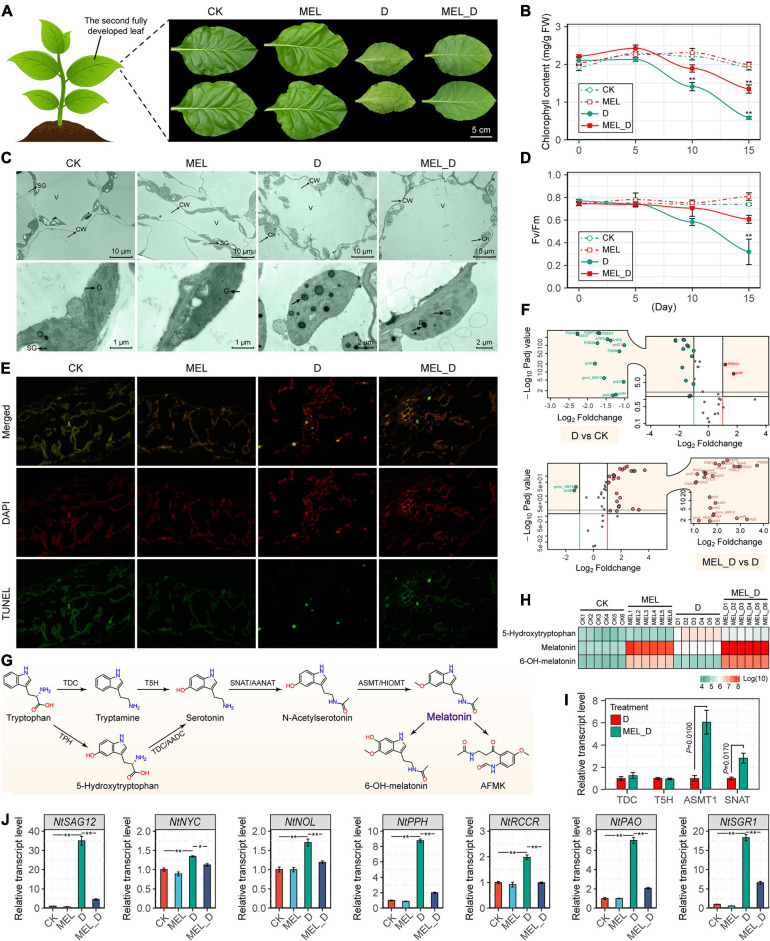
Melatonin application delays leaf senescence of *N. tabacum* subjected to dehydration stress. **(A)** Senescence phenotypes of the fourth leaves of control and melatonin-treated plants after the 15-day dehydration treatment. **(B,C)** Changes in *F*_v_/*F*_m_ and total chlorophyll contents in response to dehydration stress (*n* = 3; *P < 0.05 and **P < 0.01 compared with those in CK, according to Tukey’s test). **(D)** Ultrastructure of chloroplasts in leaves of control and melatonin-treated seedlings. Ch, chloroplast; CW, cell wall; SG, starch grain; V, vacuole; G, granal thylakoids; P, plastoglobuli. **(E)** TUNEL assay of leaf cell death. **(F)** Volcano plot showing the expression profiles of photosynthetic genes. The *X*-axis shows the log2 fold change expression; the *Y*-axis show significant differences at *P*-values (log10 transformation) < 0.05. Red and green dots represent upregulated expression of genes (multiples vary more than two times and *P*-value < 0.05) and downregulated expression of genes, respectively. **(G)** Melatonin biosynthesis pathway. **(H)** Changes in the levels of endogenous melatonin and derivatives in *N. tabacum* leaves. **(I)** Transcript levels of melatonin biosynthetic genes under water-deficit conditions. **(J)** Expression of genes responsible for leaf senescence. The vertical bars indicate SE (*n* = 3). *P < 0.05, **P < 0.01.

After water was withheld for 15 days, the leaves showed obvious yellowing (Figure 2A) and the total chlorophyll content in those plants was dramatically lower than that in the well-watered controls (Figure 2B). MEL-treated plants clearly showed retardation of dehydration-inducible leaf senescence, as shown by a decrease in chlorophyll and *F*_v_/*F*_m_ on days 10 and 15 (Figures 2B,C). Additionally, the anatomical observation of the control and MEL-treated leaves indicated that mesophyll cell density was lower in D than in MEL_D (Supplementary Figure 5), resulting in a stay-green phenotype in MEL_D-treated leaves. Further, transmission electron microscopy showed that treatments with dehydration severely disrupted the thylakoid membranes, reduced the starch grain quantities, and increased the osmiophilic granules in the chloroplast. However, the chloroplast ultrastructure in the MEL_D-treated leaves was similar to that in the unstressed leaves (Figure 2D), showing that MEL protected chloroplasts. TUNEL staining indicated that dehydration-inducible cell death was relieved by exogenous MEL application (Figure 2E). Using transcriptomic analysis, we found that the expression of most photosynthesis-associated transcripts was dramatically reduced in leaves of D compared with those of CK, whereas these genes displayed higher transcript levels in MEL_D than in D (Figure 2F and Supplementary Figure 5).

Moreover, we found that the endogenous levels of MEL and its derivative, 6-OH-MEL, were significantly elevated after treatment with 100 μM MEL (Figures 2G,H), and MEL supplementation also increased the expression of tobacco MEL biosynthetic genes, with *NtTDC*, *NtASMT1*, and *NtSNAT* expression in MEL-treated leaves being higher than those in non-treated leaves under dehydration stress (Figure 2I). Expression of a senescence marker (*NtSAG12*) and six chlorophyll catabolic genes (*NtNYC*, *NtNOL*, *NtPPH*, *NtRCCR*, *NtPAO*, and *NtSGR1*) was upregulated in response to dehydration stress, whereas their levels were decreased by MEL (Figure 2J).

### Metabolites Accumulate in Melatonin-Treated Leaves Under Dehydration Conditions

**FIGURE 3:**
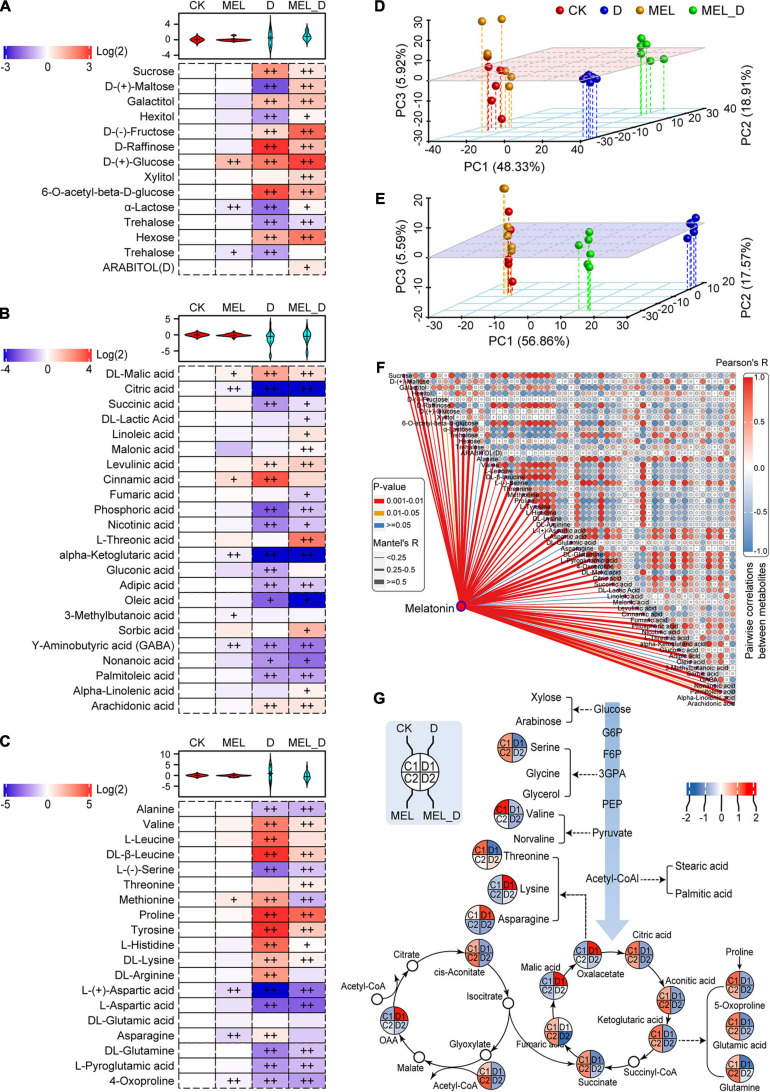
Melatonin-induced alterations in metabolite profiling of tobacco leaves exposed to dehydration stress for 15 days. **(A)** Sugar and sugar alcohol levels. **(B)** Levels of organic acids and other molecules. **(C)** Levels of amino acids and derivatives. Plus signs denote a significant metabolite accumulate difference compared with that in CK (^+^, *P* ≤ 0.05; ^++^, *P* ≤ 0.01). **(D,E)** Principal component analysis of the metabolites in the **(D)** positive and **(E)** negative ion modes. **(F)** Correlation of representative metabolites and melatonin. **(G)** Assignment of metabolites to the carbohydrate and tricarboxylic acid metabolic pathways.

To explore the impact of MEL supplementation on the metabolomic response of tobacco plants to dehydration stress, the relative abundance of metabolites was analyzed (Supplementary Figure 6 and Supplementary Tables 1–3). Principal component analysis (PCA) of all the detected metabolites showed that CK was not separated from MEL but was clearly separated from the D or MEL_D groups (Figures 3D,E). Moreover, there was a clear separation between the D and MEL_D groups. The D and MEL_D treatments induced significant accumulation of various sugars, such as sucrose, fructose, raffinose, glucose, and hexose (Figure 3A). Conversely, the accumulation of most organic acids was dramatically decreased in both D- and MEL_D-treated seedlings, including citric acid, phosphoric acid, ketoglutaric acid, adipic acid, aminobutyric acid, and palmitoleic acid (Figure 3B). Among amino acids, the levels of valine, leucine, tyrosine, histidine, and lysine were elevated in the dehydration-treated seedlings, whereas the levels of alanine, serine, aspartic acid, glutamine, and pyroglutamic acid were reduced relative to those of the controls (Figure 3C).

Next, the associations between MEL levels and the identified metabolites were investigated using correlation analysis (Figure 3F). Five metabolites were strongly positively correlated with MEL induction, which included galactitol, glucose, hexose, proline, and arachidonic acid. Contrastingly, 12 metabolites were strongly negatively correlated with MEL induction, including trehalose, alanine, serine, aspartic acid, glutamine, L-pyroglutamic acid, citric acid, phosphoric acid, ketoglutaric acid, adipic acid, GABA, and palmitoleic acid (Figure 3F). Additionally, 18 identified metabolites were assigned to carbohydrate and tricarboxylic acid (TCA) metabolic pathways (Figure 3G). The carbohydrate levels and metabolites of the TCA cycle were reduced following dehydration treatment. Among them, 12 metabolites were increased in the MEL_D group relative to those in D, such as serine, threonine, succinate, citric acid, ketoglutaric acid, glutamic acid, and glutamine. These results further confirmed that tolerance to dehydration stress may be largely dependent on carbon and amino acid metabolism.

### Comprehensive Sets of DEGs in Melatonin-Treated Leaves Under Dehydration Conditions

**FIGURE 4:**
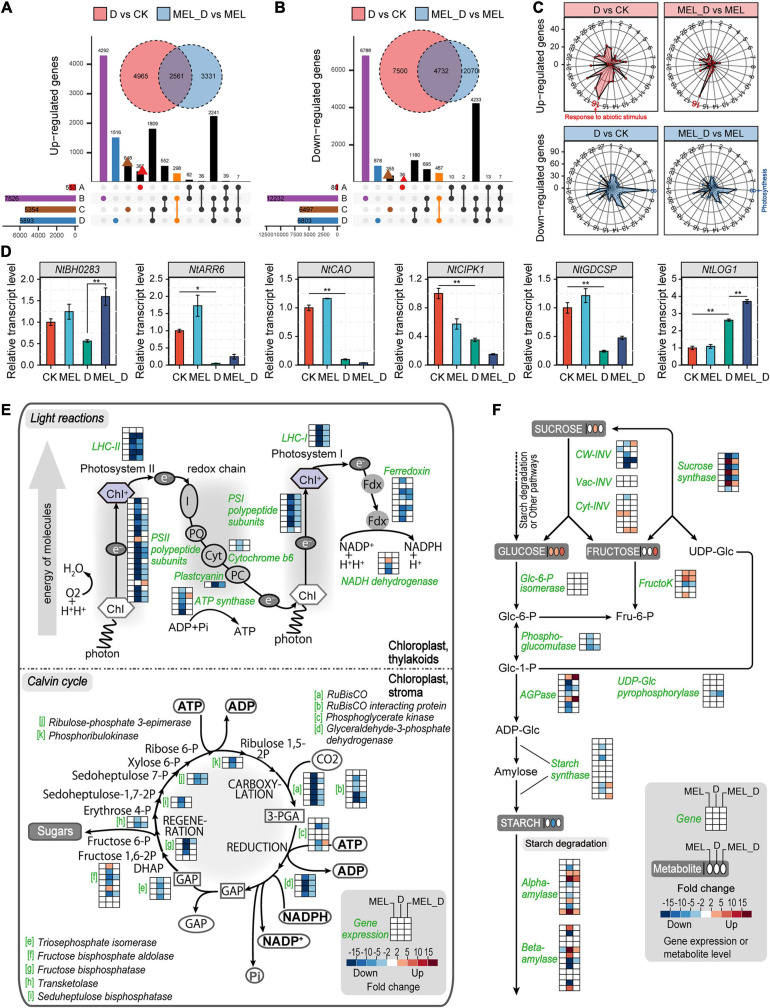
Comprehensive expression profiles of genes in tobacco leaves after dehydration stress for 15 days. **(A,B)** Comparison of the numbers of differentially expressed genes (fold change ≥ 3.0) acquired through the transcriptome analysis. Four comparison groups were analyzed, containing treatments of melatonin (MEL) versus CK **(A)**, D (dehydration) versus CK **(B)**, MEL_D (dehydration plus melatonin) versus CK **(C)**, and MEL_D versus D **(D)**. **(C)** Enriched Gene Ontology (GO) terms of DEGs. Radar charts of 27 upregulated and downregulated GO classes showing the frequency of each class. 1, carbohydrate metabolism; 2, energy metabolism; 3, lipid metabolic process; 4, nucleotide metabolism; 5, amino acid metabolism; 6, organic acid metabolism; 7, signal transduction; 8, photosynthesis; 9, DNA replication; 10, cell redox homeostasis; 11, defense response; 12, cell wall organization; 13, response to hormone; 14, response to stress; 15, response to oxidative stress; 16, response to abiotic stimulus; 17, pigment metabolic process; 18, polysaccharide metabolic process; 19, small molecule catabolic process; 20, pectinesterase activity; 21, oxidoreductase activity; 22, helicase activity; 23, peroxidase activity; 24, ribonuclease activity; 25, hydrolase activity; 26, protein ubiquitination; 27, vitamin binding. **(D)** Expression levels of the selected genes using qPCR analysis (*n* = 3). **(E)** Alterations in the expression of genes related to light and dark reactions of photosynthesis. **(F)** Alterations in the expression of genes related to starch metabolism. Pathway diagrams in **(E,F)** were constructed using MapMan.

Transcriptomic analysis identified more than 30,000 differentially expressed genes (DEGs) in MEL-, D-, and MEL_D-treated leaves versus CK (Supplementary Tables 4–6). Compared to the transcripts of untreated leaves, D-treated leaves had 7,525 and 12,232 upregulated and downregulated genes, respectively. Compared to D samples, MEL_D-treated samples had 8,111 and 4,989 upregulated and downregulated genes, respectively. Venn diagrams revealed that the number of genes commonly downregulated in D was greater than that in MEL_D (Figures 4A,B). Although a wide array of GO terms were commonly or differentially enriched among the analyzed sets, photosynthesis was the most enriched GO term, mainly because of the large number of corresponding enzymes being downregulated upon dehydration treatment (Figure 4C). The qPCR analysis of six randomly selected genes (*NtBH0283*, *NtARR6*, *NtCAO*, *NtCIPK1*, *NtGDCSP*, and *NtLOG1*) confirmed the accuracy of RNA-seq (Figure 4D). The transcription of most genes involved in photosynthetic light reactions and the Calvin cycle in D was decreased compared to those in MEL_D (Figure 4E). *Cyt*-*INV* and sucrose synthase, which are responsible for sucrose degradation, were upregulated in the D and MEL_D treatments (Figure 4F), and the AGPase and starch synthase genes, which encode enzymes for starch biosynthesis, were highly expressed in MEL_D. However, the expression of five AGPase and four starch synthase genes was reduced in D. Compared to that in CK, the expression of most genes encoding alpha- and beta-amylases, which are key enzymes involved in the degradation of starch, was not changed in MEL_D, whereas the transcription of four alpha-amylases and four beta-amylases was significantly increased in D. The expression patterns of genes related to starch degradation agreed with the decrease in starch under D treatment and its accumulation under MEL_D treatment conditions.

### Expression of Genes Involved in Phytohormone Biosynthesis in Melatonin-Treated Leaves Under Dehydration Conditions

**FIGURE 5:**
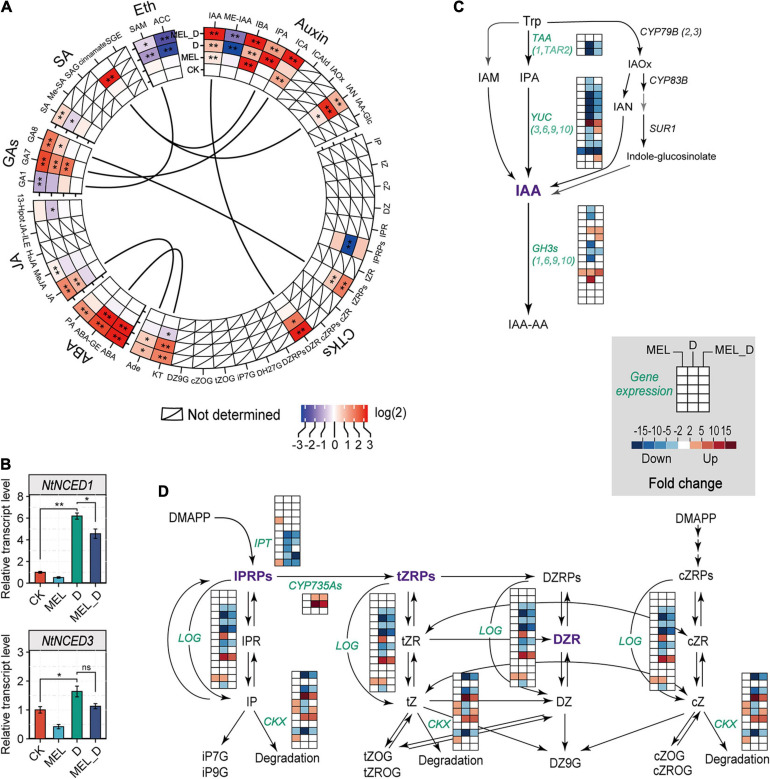
Melatonin effects on the expression of phytohormone biosynthetic genes in tobacco leaves after exposure to dehydration. **(A)** Relative levels of phytohormones based on the untargeted analysis. CTKs, cytokinins; ABA, abscisic acid; JA, jasmonates; GAs, gibberellins; SA, salicylic acid; Eth, ethylene. Asterisks symbols represented statistically significant differences (*P < 0.05, **P < 0.01). **(B)** ABA biosynthesis and the expression levels of genes encoding NCED, as determined by qPCR. Values have been presented as mean ± SE (*n* = 3). **(C)** Transcript levels of genes involved in IAA biosynthetic pathway. **(D)** Expression levels of genes related to cytokinin biosynthesis and metabolism.

To determine whether phytohormones were involved in the regulation of MEL-mediated dehydration tolerance, we analyzed the endogenous levels of various phytohormones based on the metabolome results. The levels of auxin-related molecules, specifically IAA, IBA, IPA, ICA, and IAN, were increased in the leaves under D and MEL_D conditions (Figure 5A). Similar to those of auxin-related molecules, the levels of tZRPs, DZR, and KT accumulated specifically in the D- and MEL_D-treated plants. Marked increases in ABA accumulation were observed in the D- and MEL_D-treated leaves, and the JA, GA7, and GA8 levels were also elevated in the leaves treated with D- and MEL_ D. Contrastingly, the level of 1-aminocyclopropane-1-carboxylic acid (ACC), i.e., a precursor in ethylene synthesis, was decreased in D- and MEL_D. Then, we analyzed the transcript levels of *NtNCED1* and *NtNCED3*, which encode the key enzymes involved in ABA biosynthesis, using qPCR. The expression of both genes was upregulated in leaves under D- and MEL_D conditions (Figure 5B), showing a correlation between the accumulation of ABA and the transcription of these genes. Conversely, the expression of most genes encoding the key enzyme for IAA biosynthesis, YUC, was downregulated upon D- and MEL_D treatments (Figure 5C). We also examined the alterations in expression of genes involved in cytokinin degradation (cytokinin oxidase/dehydrogenases, CKXs) and those responsible for the formation of IAA amino acid conjugates (GH3s). The expression of most CKX and GH3 genes was downregulated in D compared to that in MEL_D (Figure 5D). Further, extensively modulated expression occurred in the GA, SA, and ethylene biosynthesis pathways (Supplementary Figure 7). These data indicated that the alterations in phytohormonal levels in the MEL-, D-, and MEL_D-treated leaves were partially caused by the transcriptional regulation of genes involved in their biosynthesis and catabolism.

### Expression of Genes Involved in Senescence-Related Pathways in Melatonin-Treated Leaves Under Dehydration Conditions

**FIGURE 6:**
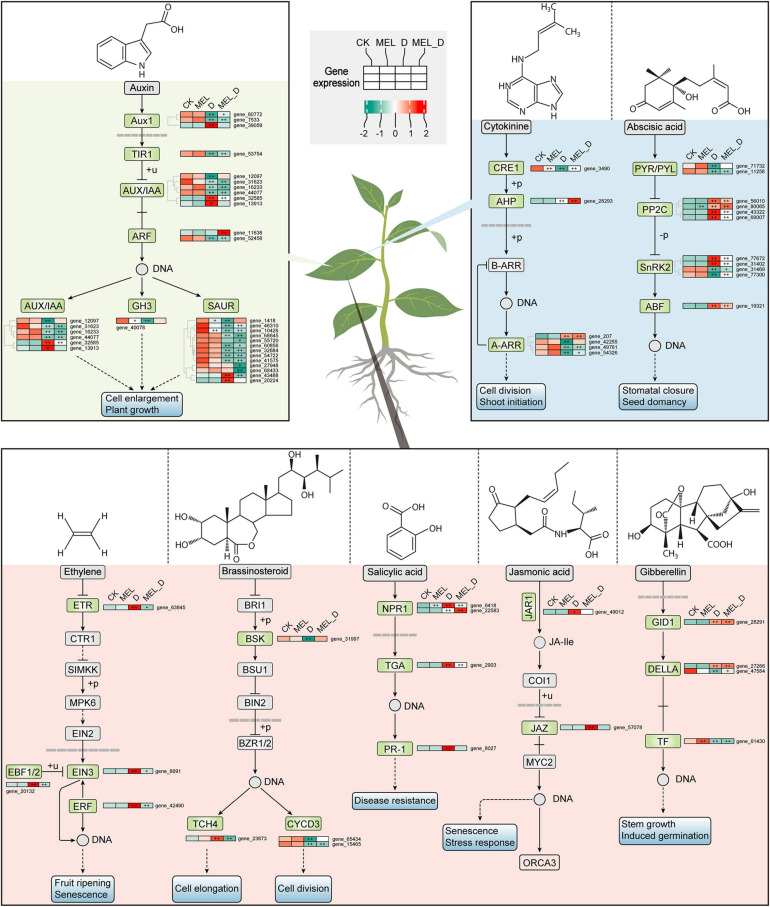
Melatonin effects on expression of genes involved in hormone signaling pathways under dehydration stress. Plus signs denote a significant gene expression difference compared with that in CK (^+^*P* ≤ 0.05, ^++^*P* ≤ 0.01).

To examine the expression profiles of genes related to senescence progress under dehydration stress, several enriched metabolic pathways were analyzed, including hormone signaling, porphyrin and chlorophyll metabolism, and regulation of autophagy. Among the genes involved in hormone transduction, 62 DEGs were enriched, including 0, 55, and 35 genes dramatically expressed in MEL, D, and MEL_D relative to those in CK, respectively (Figure 6). Except for some irregular gene expression, most genes showed downregulated expression from CK to D, such as *ARF16*, *SAUR50*, *PYL2*, *LAX5*, *IAA27*, *CYCD3*, whereas few genes showed upregulated expression upon dehydration treatment, and of special concern were the ABF and ERF genes. Furthermore, the expression of 32 and 28 genes associated with porphyrin and chlorophyll metabolism, respectively, was suppressed after D and MEL_D treatment, respectively (Supplementary Figure 8A). Additionally, 18 out of 23 genes participating in the regulation of autophagy were highly expressed in D-treated leaves, whereas the transcription of these genes in MEL_D was more downregulated than those in D (Supplementary Figure 8B), indicating that the autophagy increase induced foliar senescence. The significant genes mostly existed in D-treated leaves, and a part of them in MEL_D, indicating the extent of senescence.

### Phytohormone Profiles of Melatonin-Treated Leaves Under Dehydration Conditions

**FIGURE 7:**
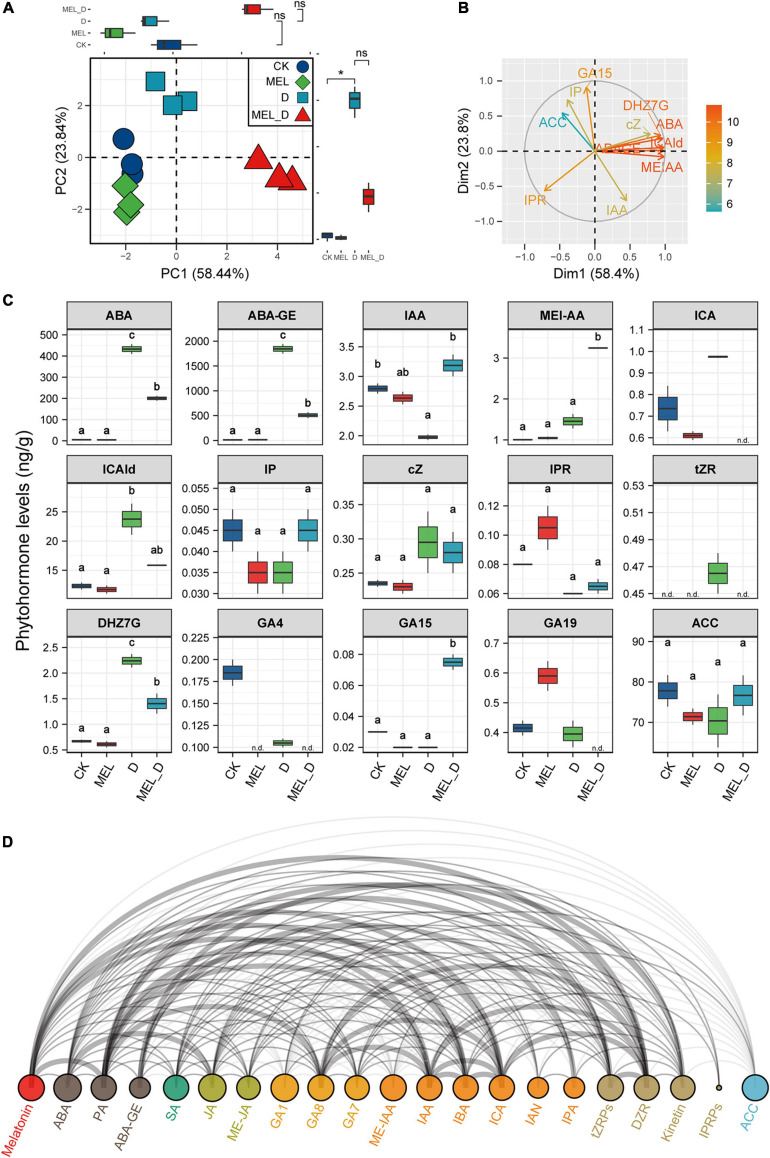
Melatonin supplementation effect on endogenous phytohormone levels. Plant hormone contents were measured in tobacco leaves subjected to four treatments: CK, control; MEL, melatonin; D, drought, and MEL_D, melatonin and drought. **(A,B)** Principal component analysis of phytohormones. PC1 and 2 have been represented in the *x*- and *y*-axis, respectively. **(C)** Statistical analysis of representative phytohormones. n.d., not detected. Data were obtained from three replicated experiments (*n* = 3), and different small letters indicated significant differences (*P* < 0.05) among the treatments. **(D)** Arc diagram illustrating the correlation between representative phytohormones.

We identified 15 phytohormones in tobacco leaves and performed PCA to analyze their responses (Figures 7A–C). The cumulative contribution ratio of the first two PCA axes was 82.28%. PC1 represented increases in phytohormones levels upon dehydration treatment, and the highest PC1 score was found in MEL_D-treated samples. Meanwhile. PC2 reflected the differences in MEL action under different growth conditions, and the PC2 values were positive for D, negative for MEL, and near zero for CK. We focused on representative phytohormones by comparing PCA loadings (Figure 7B). The highest PC1 loading was for ABA or ABA-GE, and the levels of both compounds were markedly increased in response to dehydration relative to those in well-watered leaves, while being higher in D than in MEL_D. The lowest PC1 loading was observed for IPR. IPR content in MEL-treated leaves was higher than that in untreated leaves under both normal and dehydration conditions (Figure 7C). The first- and second-highest PC2 loadings were for GA15 and IP, respectively. The accumulation of both compounds in MEL_D was increased compared with that in D. ACC displayed the second-lowest PC2 loading, without differences among the four treatments. Furthermore, we investigated the MEL-hormone relationships in plants at metabolic levels using correlation analysis (Figure 7D), with five phytohormones being strongly correlated with MEL accumulation, which included DZR, ICA, IBA, IAA, and PA. These data suggested that MEL played a prominent role in the regulation of phytohormone levels in tobacco seedlings under dehydration conditions.

### Carotenoid Profiles of Melatonin-Treated Leaves Under Dehydration Conditions

**FIGURE 8:**
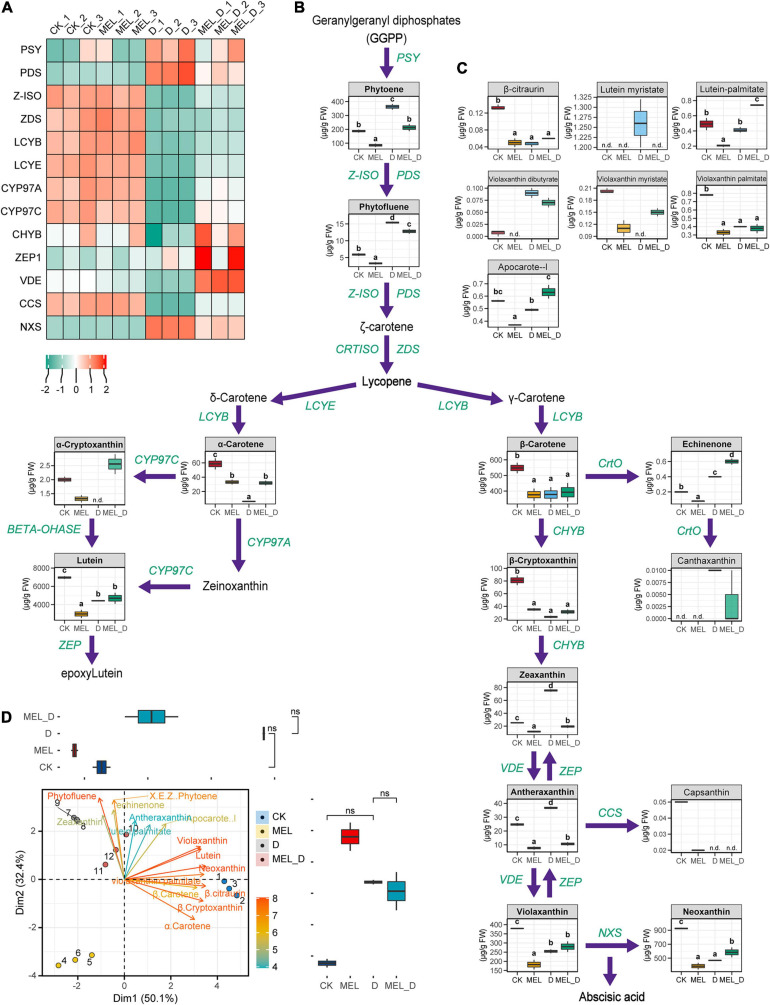
Melatonin supplementation effects on the carotenoid levels and expression of carotenoid synthesis genes. **(A)** Expression of genes involved in carotenoid biosynthesis. Green and red represent low and high expression, respectively. **(B,C)** Carotenoid identification in the leaves under four treatment conditions (CK, control; MEL, melatonin; D, drought, and MEL_D, melatonin and drought). n.d., not detected. Different letters mentioned above columns denote significant differences at *P*-values < 0.05. **(D)** Principal component analysis for carotenoids levels. Values on the *x-* and *y*- axes represent PC1 and PC2, respectively.

Data demonstrated that carotenoid synthesis was suppressed after the onset of dehydration, with accumulation of mainly phytoene, phytofluene, zeaxanthin, antheraxanthin, and violaxanthin dibutyrate (Figures 8B,C). Upon dehydration treatment, MEL-treated leaves exhibited higher levels of α-carotene, lutein, β-cryptoxanthin, violaxanthin, neoxanthin, and echinenone than untreated leaves. Specifically, phytoene accumulation in the four treatments reached 73.3–383.0 μg/g FW, with the highest content found in D and MEL_D. After phytoene, the carotenoid synthesis is divided into two branches: δ- and γ-carotene. In the δ-carotene branch, the levels of α-carotene and lutein in CK and MEL_D were markedly higher than those in MEL and D. Relative to those of the δ-carotene branch, the carotenoids in the γ-carotene branch displayed more complex accumulation patterns in the four treatments. β-carotene levels in CK and MEL_D were slightly higher than those in MEL and D, respectively. Meanwhile, yellow xanthophylls (β-cryptoxanthin, violaxanthin, and neoxanthin) mainly accumulated in CK and MEL_D. Intriguingly, we detected capsanthin only in unstressed leaves, whereas canthaxanthin was only detected under dehydration conditions (Figure 8B). Accordingly, expression profiling revealed that MEL selectively induced a different set of carotenoid biosynthesis genes from those mediated by dehydration (Figure 8A).

Then, we performed PCA to compare carotenoid levels, which indicated that neoxanthin was positively associated with violaxanthin and lutein on PC1 and negatively associated with phytofluene on PC2. Three of these traits were found with higher magnitude because of their longest vector length among all of the traits, and hence contributed the most to overall variation (Figure 8D). These changes suggested that MEL could induce the differential accumulation of carotenoids in tobacco, which contributed to MEL-treated and untreated plants displaying varying differences in the senescence process under dehydration conditions.

### Melatonin-Mediated Transcriptional Reprogramming in Response to Dehydration Stress

**FIGURE 9:**
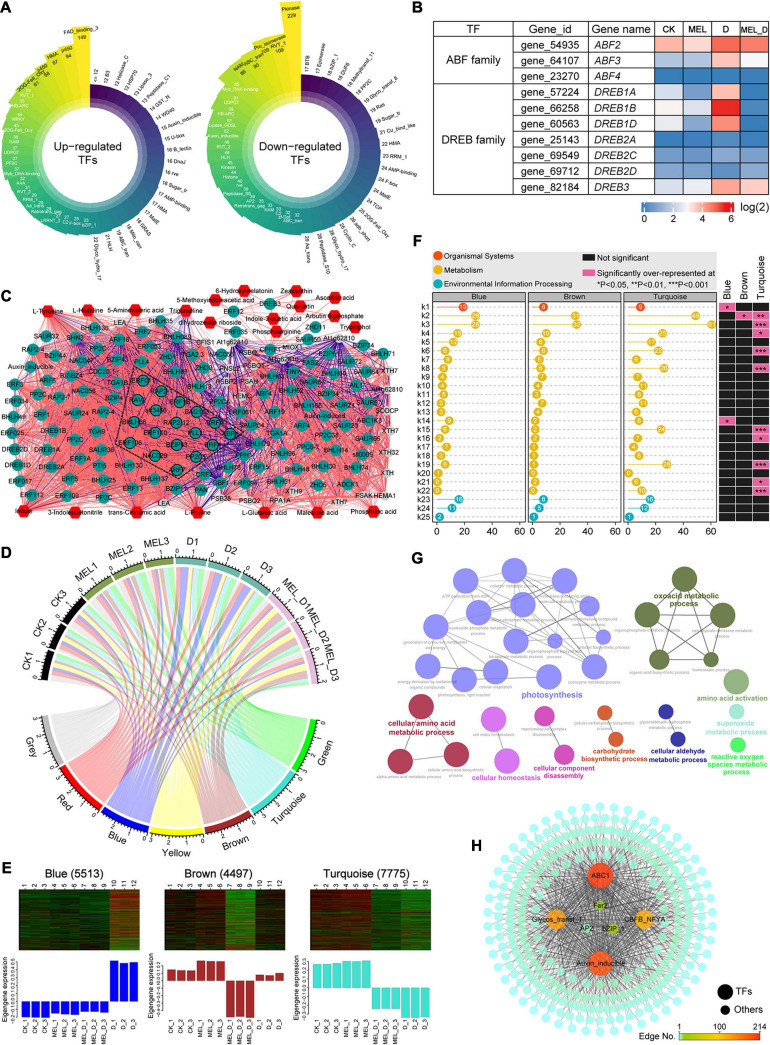
Transcriptional modulation regulated by melatonin in tobacco plants exposed to dehydration conditions. **(A)** Classification of transcription factor families in upregulated and downregulated genes expressed under dehydration stress. **(B)** Expression levels of ABA-responsive element-binding (AREBs) and dehydration-responsive element-binding (DREBs) genes. **(C)** Transcript–metabolite correlation network. Different node colors represent TF genes (green), metabolic genes (gray) and metabolites (red). Red and blue edges indicate positive and negative correlations, respectively. Edge thickness indicates the correlation coefficient value for each correlated pair. **(D)** Module-sample associations. Edge thickness represents the correlation coefficient between modules and samples. **(E)** Eigenprotein expression profile for three specific modules (blue, brown, and turquoise) in different samples. The number of genes for selected modules is mentioned in parenthesis. **(F)** KEGG pathway analysis of gene lists from the key modules. k1, plant-pathogen interaction; k2, biosynthesis of amino acids; k3, carbon metabolism; k4, glycolysis/Gluconeogenesis; k5, starch and sucrose metabolism; k6, carbon fixation in photosynthetic organisms; k7, arginine and proline metabolism; k8, glyoxylate and dicarboxylate metabolism; k9, phenylpropanoid biosynthesis; k10, alanine, aspartate and glutamate metabolism; k11, fatty acid biosynthesis; k12, phenylalanine, tyrosine and tryptophan biosynthesis; k13, phenylalanine metabolism; k14, ascorbate and aldarate metabolism; k15, porphyrin and chlorophyll metabolism; k16, carotenoid biosynthesis; k17, tryptophan metabolism; k18, nitrogen metabolism; k19, photosynthesis; k20, zeatin biosynthesis; k21, nicotinate and nicotinamide metabolism; k22, photosynthesis-antenna proteins; k23, plant hormone signal transduction; k24, MAPK signaling pathway-plant; k25, ABC transporters. **(G)** Enriched GO terms for the turquoise module. **(H)** Co-expression network analysis of the turquoise module with edge weight over 0.6.

To further elucidate the underlying mechanism by which MEL regulates the dehydration response, we analyzed the transcriptional regulatory networks. In our transcriptome data, 5,741 TF genes were differentially expressed in response to dehydration stress, including AP2, WRKY, bZIP, NAC, PP2C, bHLH, and MYB family members (Figure 9A). The heat map showed that the 10 ABA-responsive element-binding (AREB)/ABF and DREB members exhibited varying expression patterns (Figure 9B). As a first step to understand the networks of MEL-mediated transcriptional reprogramming, we first constructed a correlation network based on the expression of genes and metabolites (Figure 9C). In this network analysis, differentially expressed TF genes and key transcripts were mainly selected from clusters 1 and 4 (Supplementary Figure 9 and Supplementary Table 7) and the metabolites were screened. The correlated pairs were filtered using a correlation coefficient of *r* > 0.99. A transcript–metabolite correlation network including 173 nodes and 3,920 edges was visualized in Cytoscape, indicating that numerous core TF genes play a central role in the regulation of dehydration responses. Additionally, gene co-expression network analysis (WGCNA) identified seven modules, as shown in the gene dendrogram (Supplementary Figure 10). Furthermore, we associated each of the co-expression modules with 12 treatment samples using correlation analysis (Figure 9D), showing that the turquoise module showed a relatively high correlation with the dehydration treatment. The eigengene expression for the co-expression modules revealed that several turquoise module genes were weakly expressed in the D and MEL_D treatment groups (Figure 9E). In the turquoise module, KEGG pathway and GO enrichment analyses indicated that gene sets related to photosynthesis, porphyrin/chlorophyll metabolism, carbon metabolism, glyoxylate/dicarboxylate metabolism, organic acid metabolic process, amino acid metabolism, and cellular homeostasis were over-represented (Figures 9F,G). The gene sets for the turquoise module were primarily downregulated upon dehydration treatment and were highly correlated with ABC1, auxin inducible, CBF, bZIP, AP2, and Fer2 (Figure 9H), which appear to be candidate genes that may regulate tobacco defenses to dehydration stress. These results indicate that MEL-mediated transcriptional networks regulate leaf senescence and the dehydration response in tobacco.

## Discussion

In this study, we discovered that MEL application promoted tobacco seedling resistance to prolonged dehydration exposure through alterations in the metabolic flux into photosynthesis, phytohormone biosynthesis, carbohydrate metabolism, and carotenoid synthesis, subsequently mitigating leaf senescence. Our results also reveal that the molecular basis by which MEL delays leaf senescence involves the translational regulation of stress response, chlorophyll degradation, and hormone signaling in stressed plants.

### Melatonin Confers Enhanced Resistance to Dehydration by Modulating Redox Homeostasis

Plant stress tolerance is tightly associated with the activation of internal ROS-scavenging systems, consisting of low-molecular weight antioxidants and antioxidant enzymes (Mittler, 2002). Under drought conditions, elevated oxidation levels would lead to either enhanced sustainability or ultimately cell death, depending on the intensity of the oxidative signal activated. High ROS concentrations that surpass the capacity of the detoxifying machinery may activate apoptosis-inducing factors, thereby causing cell death (Miller et al., 2010; Khan et al., 2021). The initial MEL function in organisms is to act as a free radical agent to eliminate reactive oxygen and nitrogen species and protect plant cells from oxidative damage (Arnao and Hernández-Ruiz, 2019). Exogenous application of MEL significantly reduces ROS accumulation and lipid peroxidation, as well as enhances the antioxidant system in apple (Wang et al., 2013a), cucumber (Zhang et al., 2014), grape (Meng et al., 2014), bermudagrass (Shi et al., 2015b), and *Carya cathayensis* (Sharma et al., 2020). Similar results were obtained in this study, which showed that dehydration stress increased the occurrence of ROS-induced oxidative stress in tobacco plants, but MEL treatment reduced ROS accumulation and alleviated plant growth inhibition (Figure 1). Accordingly, MDA and EL, as important indicators of oxidative damage, were relatively less pronounced in MEL-treated stressed plants (Figures 1D,E), further implying that MEL might protect cell membranes against oxidative stress. Moreover, MEL-activated antioxidant defenses play key roles in tobacco resistance to water deficit. This study suggested that MEL treatment increased the enzymatic and non-enzymatic antioxidant contents and upregulated mRNA levels of anti-stress genes in stressed tobacco leaves (Supplementary Figures 3, 4), which was consistent with the decrease in ROS levels. In this context, the coordinated regulation of multiple antioxidative enzymes and antioxidants efficiently scavenged excessive ROS, thereby maintaining the cellular redox state in stressed plants. Thus, MEL-treated plants achieve high tolerance to dehydration-induced oxidative damage through the development of a powerful detoxifying capacity, which in turn results in better growth behavior under stress conditions.

### Melatonin Delays Early Leaf Senescence and Inhibits Chlorophyll Breakdown

As a potent anti-senescent compound, MEL plays a critical role in inhibiting premature leaf senescence, as reported by Wang et al. (2013b), who demonstrated that long-term MEL treatment induced a marked delay in leaf senescence of *Malus hupehensis* by regulating metabolic status and protein degradation. In this study, we found that MEL-treated tobacco leaves exhibited a delayed senescence phenotype, including an increase in photosynthetic rate (Figure 2). Moreover, leaf MEL content was slightly elevated with dehydration, and MEL addition had a significant effect in maintaining high levels of *in vivo* MEL concentration (Figure 2H). Accordingly, the expression of MEL biosynthetic genes, such as *NtTDC*, *NtT5H*, *NtASMT1*, and *NtSNAT* was upregulated by MEL treatment (Figure 2I). Similarly, a recent study in citrus trees also showed that the transcript levels of MEL biosynthetic genes, *CsTDC1*, *CsT5H*, and *CsASMTs* were upregulated by exogenous MEL supplementation (Nehela and Killiny, 2020). Further, endogenous MEL level is remarkably induced in MEL-pretreated *Medicago sativa* plants subjected to prolonged drought stress (7 days) (Antoniou et al., 2017). Chlorophyll degradation progresses throughout senescence but is promoted upon drought stress. Our data clearly demonstrated that external administration of MEL restored the dehydration-stressed plants’ leaf yellowing phenotype to levels comparable to those of controls, while maintaining a higher photosynthetic efficiency and chlorophyll level, and preventing the breakdown of chloroplast ultrastructure (Figure 2). These changes are in line with previous reports suggesting that drought-induced chlorophyll degradation was attenuated by MEL application in apples (Wang et al., 2013b) and Chinese hickory (Sharma et al., 2020). Similarly, our work indicated cell death levels were decreased in MEL-treated stressed seedlings (Figure 2E), implying that MEL can ameliorate dehydration-induced foliar senescence by decreasing cell death. Upon induction of dehydration stress, the expression of several senescence-related genes is modified. In this experiment, exogenous MEL strongly mitigated the upregulated expression of senescence markers and chlorophyll catabolic genes (Figure 2J), which are responsible for the degradation of chlorophyll (Wang et al., 2012). These findings suggest another mechanism by which MEL preserves chlorophyll levels, increases leaf longevity, and alleviates dehydration-induced photoinhibition, in addition to ROS detoxification.

### Melatonin Regulates Leaf Senescence via Modulation of Phytohormonal Biosynthesis and Signaling

In plants, previous studies on ABA signaling cascades showed that ABA induces premature leaf senescence via activating a series of senescence-associated genes (Sakuraba et al., 2020). Specifically, the ABA receptor *PYL9* was shown to accelerate leaf senescence in *Arabidopsis* (Zhao et al., 2016). Recently, overexpression of *ABFs* in tobacco plants not only induced leaf senescence and ABA production, but also activated genes that code for ABA biosynthesis and chlorophyll degradation (Tan et al., 2019). Similarly, our plant hormone profiles of tobacco leaves showed that the stress hormone ABA was the phytohormone most inducible by dehydration (Figure 7). The expression of key genes (*NtNCED1*, *NtNCED3*, and *NtABF*) involved in ABA biosynthesis and signaling was increased upon dehydration treatment (Figures 5, 6), which supported the suggestion that prolonged dehydration stress gates ABA signaling to induce leaf senescence in tobacco plants. In contrast, the expression of genes responsible for auxin biosynthesis and signaling was suppressed by dehydration stress. These results suggested that auxin and ABA antagonistically regulate dehydration-induced senescence.

There is a close relationship between phytohormone levels and MEL concentration. Accumulating evidence supports that MEL acts synergistically or antagonistically with other phytohormones such as auxin, CTK, GA, ABA, ethylene, and JA during biological processes, particularly in stress responses (Zhang et al., 2014; Arnao and Hernández-Ruiz, 2015, 2018; Fan et al., 2018; Tan et al., 2019; Nehela and Killiny, 2020), with ABA and auxin being particularly interesting. Previous studies have shown that MEL decreases the anabolic capacity for ABA biosynthesis and increases catabolic capacity for ABA metabolism, thereby regulating the functions of stomata in dehydration-treated *Malus* plants (Li et al., 2015). In drought-stressed apple leaves, MEL selectively downregulates *MdNCED3* and upregulates ABA catabolic enzymes (*MdCYP707A1* and *MdCYP707A2*), thus halving endogenous ABA production (Li et al., 2015). In agreement with these reports, our results revealed that the accumulation of ABA in stressed tobacco plants was attenuated by exogenous application of MEL, but not that of auxin, which was remarkably higher in MEL-treated stressed plants than in non-treated stressed plants (Figure 7). Similar findings were obtained in Chinese flowering cabbage, with MEL attenuating leaf senescence by inhibiting ABFs (*BrABF1*, *BrABF4*, and *BrABI5*)-mediated ABA accumulation and reducing expression of chlorophyll catabolic genes (Tan et al., 2019). The MEL-dependent defense against dehydration stress might also be associated with other hormones, including CTK, GA, JA, and ethylene, as MEL affects key gene expression involved in these signaling pathways, such as ARR3 (response regulator 3), GID1C (gibberellin receptor GID1), JAR1 (jasmonic acid-amino synthetase) and ERF1 (ethylene response factor 1) (Figure 6). These findings demonstrate that MEL supplementation decreases ABA accumulation and maintains high IAA levels by simultaneously inhibiting and activating ABA and IAA biosynthetic genes, respectively, under dehydration conditions. Thus, MEL-triggered delayed leaf senescence appears, at least in part, by modulating hormone biosynthesis and signaling. In the future, it will be important to unravel the MEL-ABA-auxin interactions and their roles in the control of plant senescence to improve our understanding of hormone networks regulating plant defense.

### Melatonin Participates in Plant Response to Dehydration Stress by Modulating the Transcriptional Network

Transcriptional regulation comprises a dynamic gene network involving the interaction of TFs with the *cis* elements of the target genes (Breeze et al., 2011). Multiple TF families have been implicated in the regulatory network underlying leaf senescence, including MYBs, WRKYs, NACs, GRAS, and NF-Ys (Balazadeh et al., 2011; Wu et al., 2012; Tian et al., 2020). Recently, NAC (NAM, ATAF1/2, and CUC2) proteins have been identified as key players in the induction of leaf senescence (Fang et al., 2008; Balazadeh et al., 2011; [Bibr B63][Bibr B40]). In *Arabidopsis*, NAP plays a key role in regulating both senescence and hormone signaling, promoting ABA biosynthesis and chlorophyll degradation by activating the expression of *AAO3* and *NYC1* (Yang et al., 2015). Similarly, *OsNAP* and *OsNAC2* accelerate leaf senescence in rice by directly activating ABA biosynthetic genes and chlorophyll degradation genes (Liang et al., 2014; Mao et al., 2017). Interestingly, several TFs such as DREB/CBF, HSF, ZFPs, WRKY, NAC, and MYB have previously been proposed as putative targets of MEL involved in stress responses (Liang et al., 2015; Shi et al., 2015c, 2018). Consistently, our transcriptomic profiling analysis revealed that the expression of a significant proportion of the senescence-controlling TFs, including 41 NACs, 4 DREBs, and 9 HSFs, as well as two ABF proteins, were transcriptionally induced in leaves by dehydration treatment but repressed by MEL (Figure 9). Furthermore, co-expression network analysis indicated that TFs such as ABC1, auxin inducible, CBF, bZIP, AP2, and Fer2 were strongly associated with leaf senescence in stressed tobacco plants by modulating photosynthesis and other metabolic processes. These results demonstrated that MEL-mediated transcriptional regulators are likely to play prominent roles in the control of leaf senescence caused by dehydration stress. Considering that MEL can regulate ROS homeostasis and that elevated ROS production triggers senescence signals, these key regulatory genes should be excellent candidates for dissecting MEL-mediated leaf longevity and stress resistance. Thus, elucidating the crosstalk between MEL and key TFs that control plant senescence under dehydration conditions is essential. Transcripts are only one part of the regulatory networks, and it is also possible that MEL regulates the progression of senescence through other molecular targets of currently unknown functions. Thus, further research focusing on defining the detailed mechanisms by which MEL controls leaf longevity during dehydration is required.

## Conclusion

**FIGURE 10:**
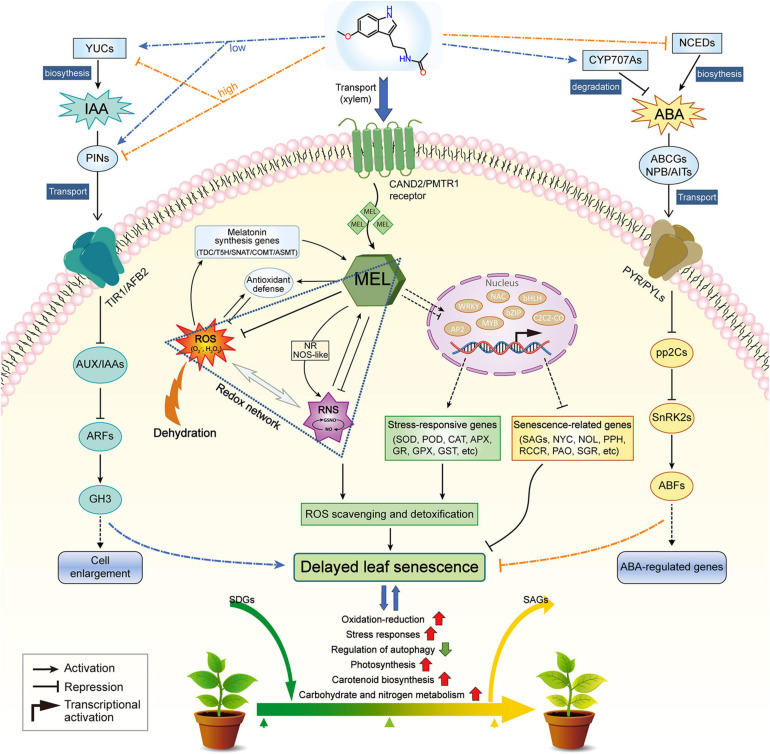
A working model depicting the role of melatonin in regulating dehydration-induced leaf senescence. This model has been proposed based on our own observations and findings reported in literature references. Red and green arrows indicate increased and decreased levels of components, respectively.

In summary, the protective effect of MEL against dehydration-induced leaf senescence points to its physiological roles as a powerful radical scavenger, promoting ROS detoxification, or as a more specific agent associated with the regulation of senescence-related genes. MEL-treated plants clearly exhibited delayed dehydration-inducible leaf senescence, as shown by less water loss, decreased lipid peroxidation, and higher photochemical efficiency, relative to that of untreated plants. In the presence of MEL, MEL regulates the transcription of TFs, such as NAC, bHLH, AP2, bZIP, MYB, WRKY, and ABF, which in turn modulates the expression of genes responsible for ROS scavenging and chlorophyll degradation. The validity of this function is manifested by our observation that MEL addition suppresses intracellular ROS production during dehydration stress. Consequently, the expression of genes involved in diverse biological functions, such as oxidation–reduction, stress responses, autophagy, photosynthesis, carotenoid biosynthesis, and carbohydrate/nitrogen metabolism, are modified by MEL, resulting in delayed leaf senescence and elevated endurance to dehydration stress (Figure 10). These findings provide evidence of MEL functioning as a positive factor in mitigating dehydration-promoted oxidative damage and leaf senescence, and highlights the exciting potential of MEL for crop improvement.

## Data Availability Statement

The original contributions generated for this study are publicly available. This data can be found here: NCBI repository, accession: PRJNA691642.

## Author Contributions

JX and ZX conceived the experiments. ZC drafted the manuscript. WJ participated in the main experiments in this work, with assistance from SL. All authors read and approved the final manuscript.

## Conflict of Interest

The authors declare that the research was conducted in the absence of any commercial or financial relationships that could be construed as a potential conflict of interest.

## Footnotes

1 www.mzcloud.org/

2 www.chemspider.com/

3 ftp://ftp.solgenomics.net/genomes/Nicotiana_tabacum/

4 https://sciex.com.cn/
