# Supplementary material for: Enhancement of Nicotiana tabacum Resistance Against Dehydration-Induced Leaf Senescence via Metabolite/Phytohormone-Gene Regulatory Networks Modulated by Melatonin
Source: Front Plant Sci. 2021 Jul 6;12:686062. doi: 10.3389/fpls.2021.686062 (PMC8291779; doi:10.3389/fpls.2021.686062)
Supplement: Supplementary file 9 [file Presentation_1.PDF]

Supplementary Material

Supplementary Figures

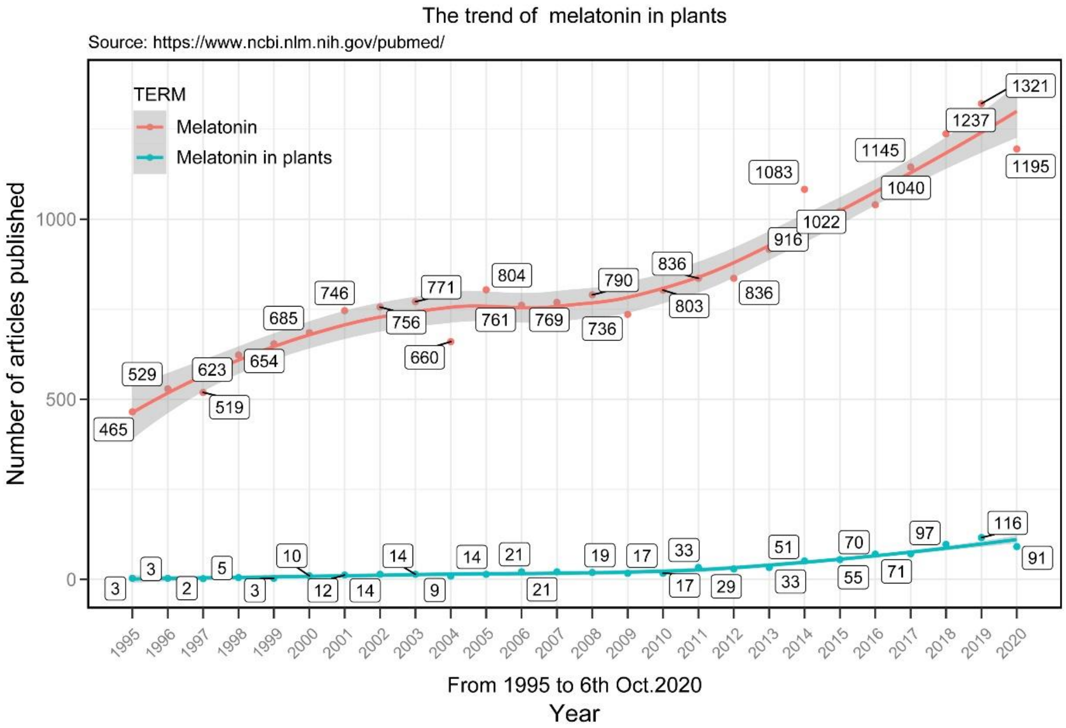

Supplementary Figure 1. Number of articles published on melatonin in plants from the year 1995 to 2020. Information was retrieved with key words "Melatonin in Plants" with the desired year bracket.

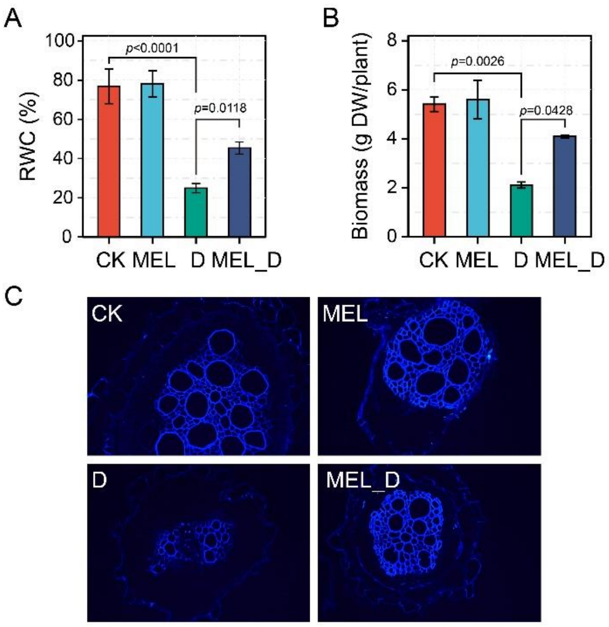

Supplementary Figure 2. Effects of exogenous melatonin on morpho-anatomical and physiological characteristics of *N tabacum* seedlings under dehydration stress. (**A–B**) Relative water content (RWC) and dry weight (DW) of the seedlings. (**C**) Casparian strip of the roots of control and melatonin-treated seedlings. Root cross sections were made at 20 mm from the apex.

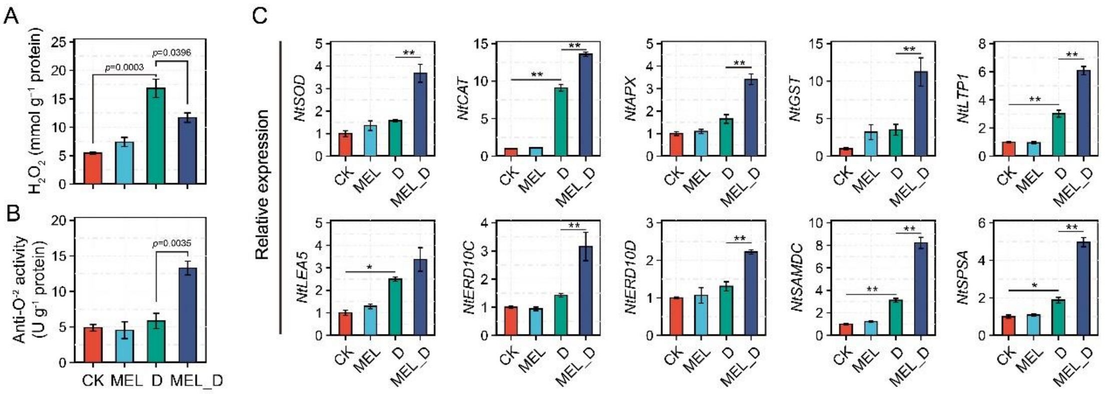

Supplementary Figure 3. Mitigation effects of exogenous melatonin on dehydration stress in *N tabacum* seedlings. (**A, B**) Quantification of $^{\bullet}O_2^{-}$ and H_2O_2 levels in tobacco leaves. (**C**) Expression analysis of stress-responsive genes in tobacco leaves. The expression level in CK was set to 1. Error bars represent the SE calculated from three independent biological replicates. *(*P* < 0.05) and **(*P* < 0.01) indicates significant differences according to an ANOVA test followed by Tukey's test.

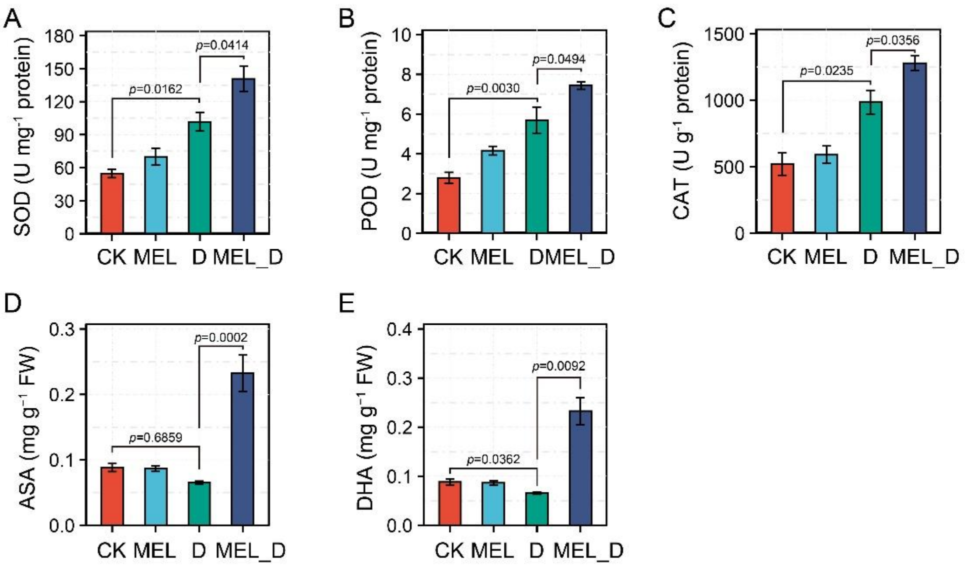

Supplementary Figure 4. Effects of exogenous melatonin on enzymatic and non-enzymatic antioxidants in *N tabacum* seedlings under dehydration stress. (**A–C**) The activities of superoxide dismutase (SOD), peroxidase (POD), and catalase (CAT) in tobacco leaves after dehydration stress. (**D, E**) Determination of ascorbic acid (AsA) and dehydroascorbate (DHA) contents. Error bars represent the SE calculated from three independent biological replicates.

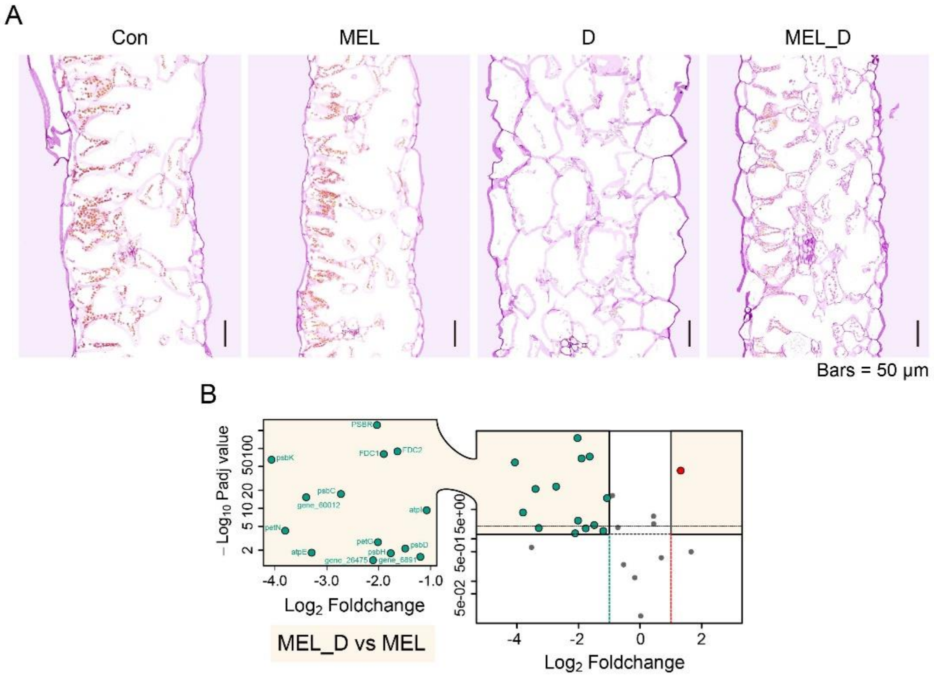

Supplementary Figure 5. The mitigation effects of exogenous melatonin on dehydration stress in *N tabacum* leaves. (**A**) Histological alterations in leaves of dehydration-stressed seedlings treated with melatonin. (**B**) Volcano plot showing the expression profiles of photosynthetic genes. The horizontal axis shows the log2 fold change expression; the vertical axis shows significant differences in *P* values (log10 transformation) of < 0.05. The red dots represent upregulated genes (multiples vary more than 2 times and *P* value < 0.05), and green dots represent downregulated genes.

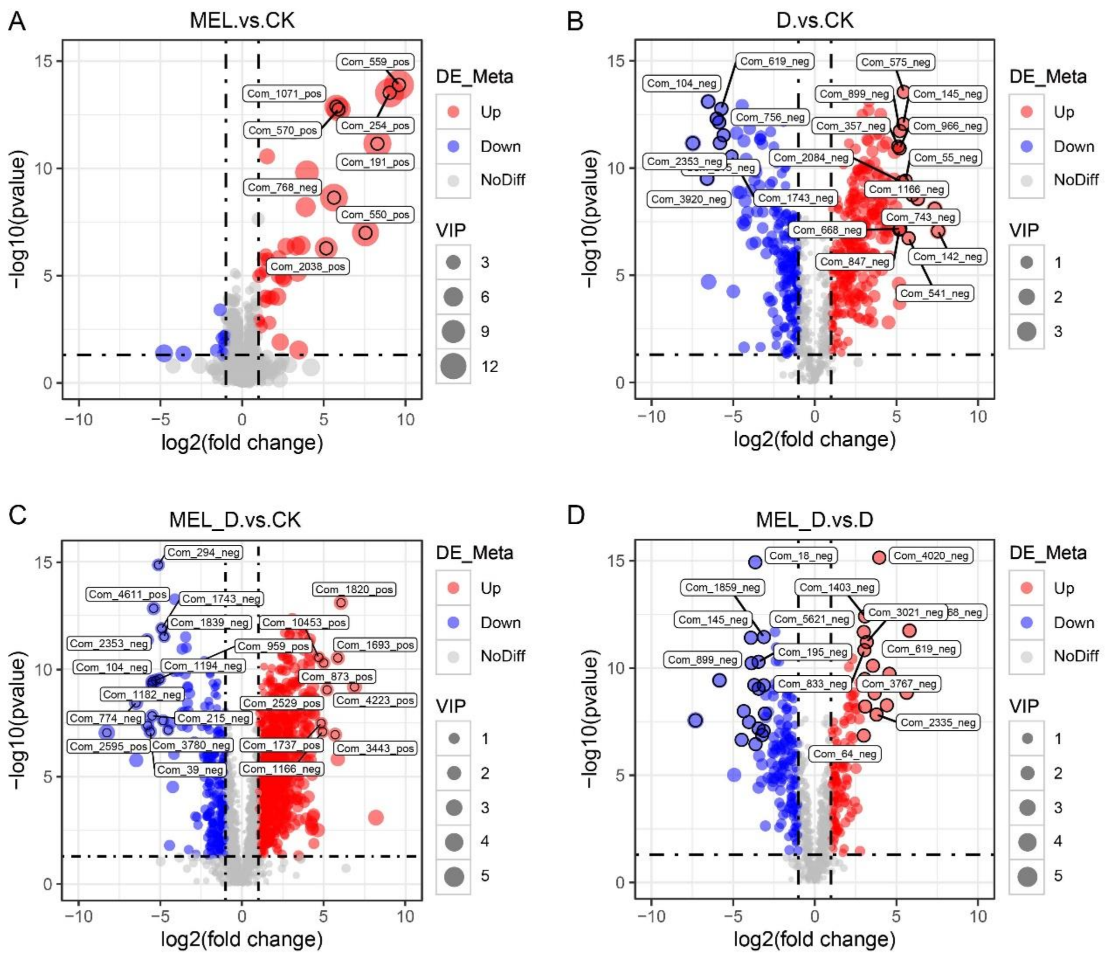

Supplementary Figure 6. Volcano plot showing the differentially expressed metabolites (fold change ⩾ 2.0; VIP ⩾ 1) acquired through the metabolome analysis. Four comparisons were conducted, including treatments of melatonin (MEL) versus CK (**A**), D (dehydration) versus CK (**B**), MEL_D (dehydration plus melatonin) versus CK (**C**), and MEL_D versus D (**D**). The horizontal axis shows the log2 fold change expression; the vertical axis shows the significant different *p*-value (log10 transformation). The red and blue dots are significant differentially abundant metabolites (multiples vary more than 2 times, *P* value < 0.05, and VIP ⩾ 1), and grey dots are no differential changes in the metabolites.

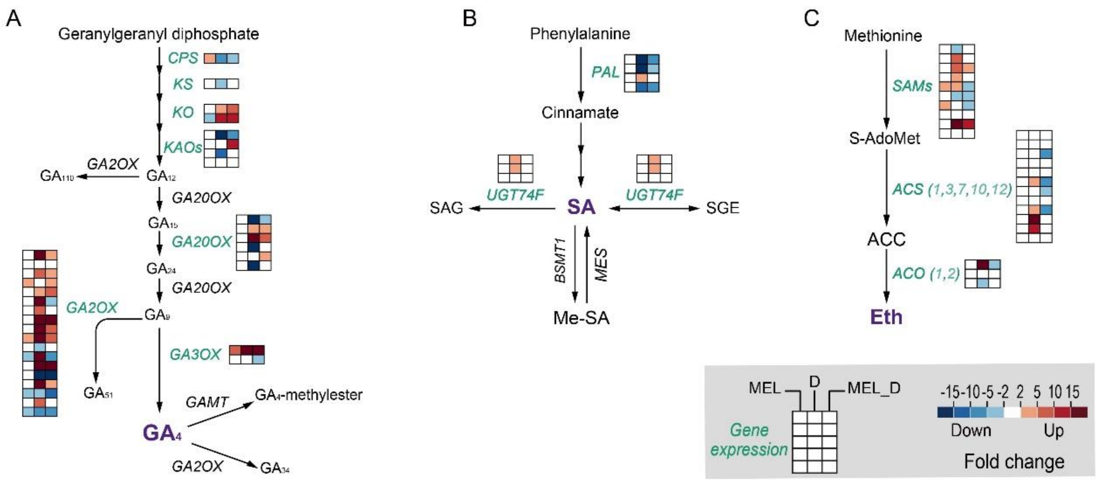

Supplementary Figure 7. Melatonin effects on the expression of phytohormone biosynthetic genes in tobacco leaves after exposure to dehydration stress. (**A–C**) Changes in the expression of genes involved in GA (**A**), SA (**B**), and ethylene (**C**) biosynthetic pathways. Blue and red represent low and high gene relative expression levels respectively.

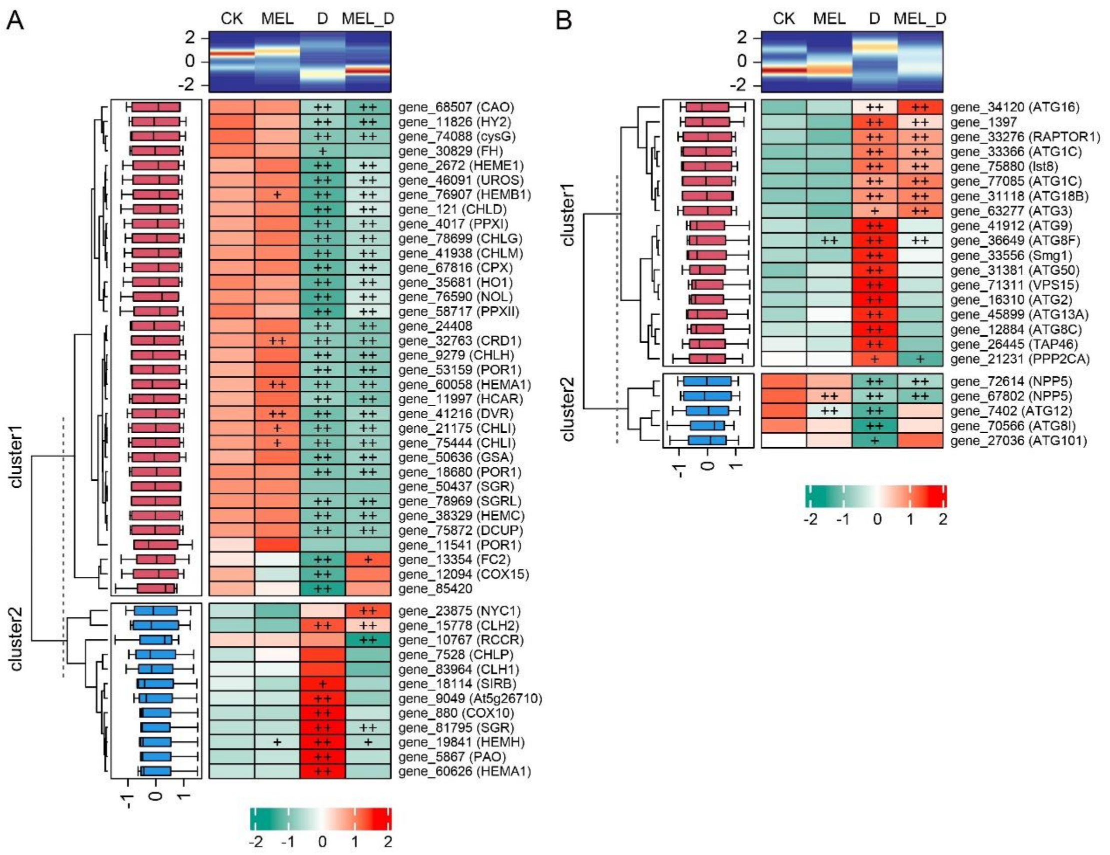

Supplementary Figure 8. Melatonin effects on expression of genes involved in senescence-related pathways under dehydration stress. (**A**) Porphyrin and chlorophyll metabolism. (**B**) Regulation of autophagy. Plus signs denote a significant gene expression difference compared with that in CK (+, $P \leq 0.05$; ++, $P \leq 0.01$). Red and green indicate high and low expression, respectively.

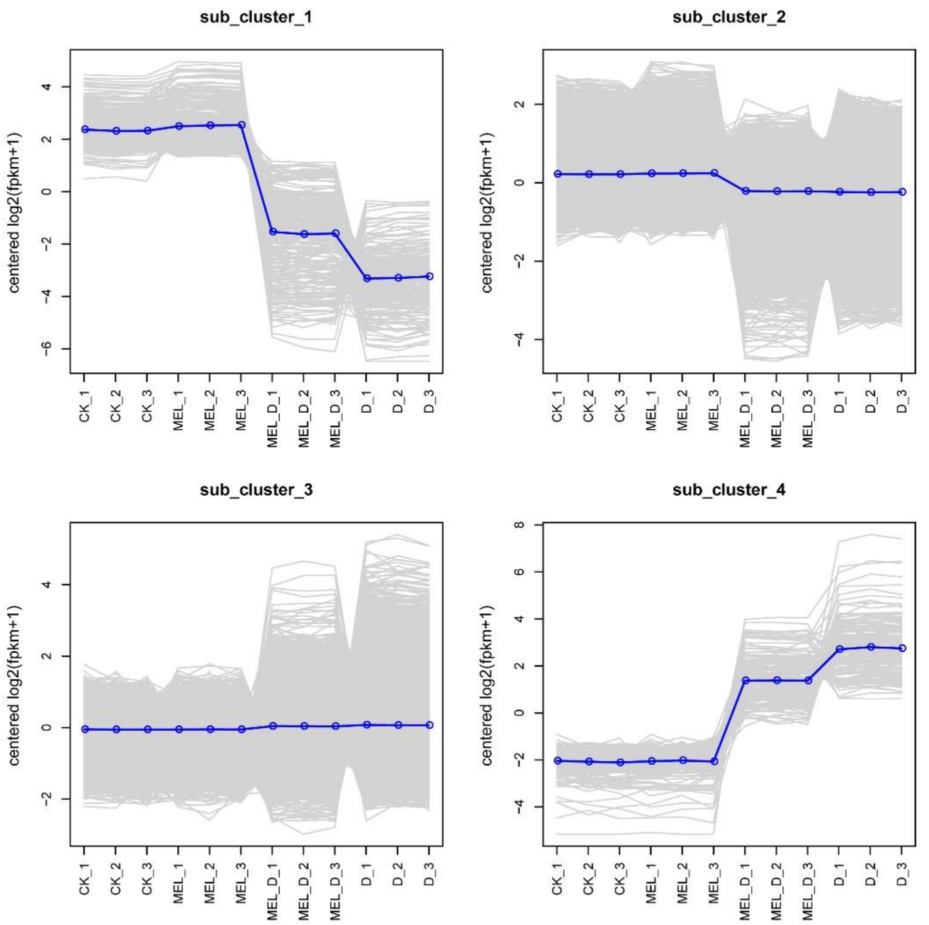

Supplementary Figure 9. Expression patterns of all differentially expressed genes. Gene expression profiles of individual genes are depicted in grey lines, and average expression profiles for each series are depicted in blue lines. Cluster 1 represents the profile of 300 transcripts that are primarily involved in photosynthesis. Cluster 4 represents the profile of 168 transcripts that are primarily related to stress responses.

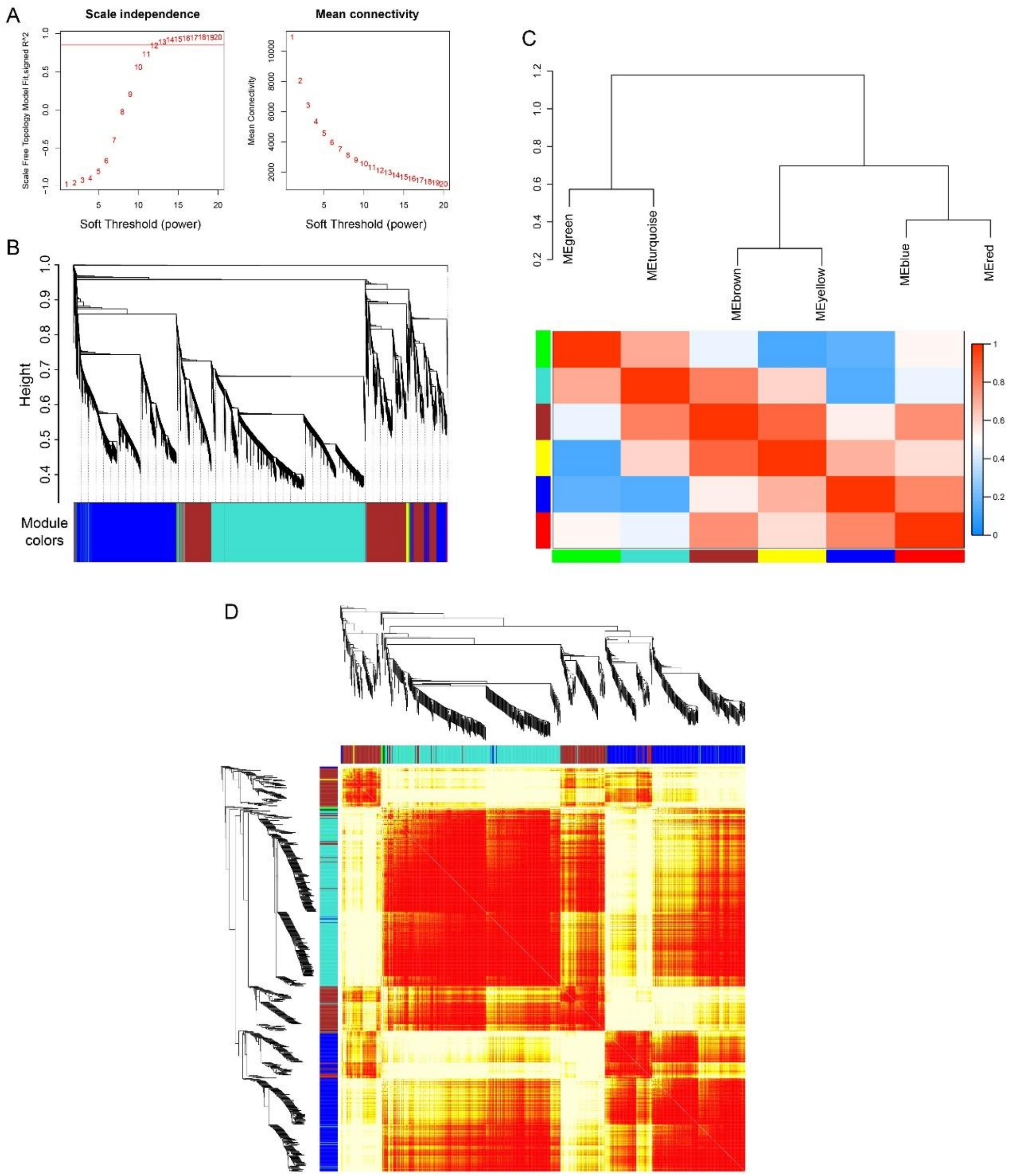

Supplementary Figure 10. Co-expression network analysis with WGCNA. (**A**) Selection of soft threshold. (**B**) Hierarchical clustering tree (dendrogram) of genes based on co-expression network analysis. Each tree branch constitutes a module and each leaf in the tree corresponds to individual gene. (**C**) Heat map showing the correlation between modules. (**D**) Network heatmap plot for selected genes.

Supplementary Tables

Supplementary Table S1. List of differentially expressed metabolites in MEL-treated leaves compared with CK.

Supplementary Table S2. List of differentially expressed metabolites in D-treated leaves compared with CK.

Supplementary Table S3. List of differentially expressed metabolites in MEL_D-treated leaves compared with CK.

Supplementary Table S4. Differentially expressed genes (DEGs) in MEL-treated leaves (MEL/CK).

Supplementary Table S5. Differentially expressed genes (DEGs) in D-treated leaves (D/CK).

Supplementary Table S6. Differentially expressed genes (DEGs) in MEL_D-treated leaves (MEL_D/CK).

Supplementary Table S7. GO categories show the most significant biological process and molecular function terms falling into clusters 1 and 4.

Supplementary Table S8. Primer sequences used for qPCR analyses.
